# Comparative regenerative mechanisms of adipose-derived mesenchymal stem cell- and conditioned medium-loaded three-dimensional bioprinted hydrogels in chronic diabetic wounds

**DOI:** 10.1093/rb/rbag095

**Published:** 2026-05-13

**Authors:** Lili Wang, Xiaoqi Li, Xinyu Zhang, Qingfei Chu, Linjie Ye, Tingting Jin, Ji Wang, Yi Sun, Sufan Wu, Ting Li

**Affiliations:** Center for Plastic & Reconstructive Surgery, Department of Plastic & Reconstructive Surgery, Zhejiang Provincial People’s Hospital, Affiliated People’s Hospital, Hangzhou Medical College, Hangzhou, Zhejiang 310014, China; Center for Plastic & Reconstructive Surgery, Department of Plastic & Reconstructive Surgery, Zhejiang Provincial People’s Hospital, Affiliated People’s Hospital, Hangzhou Medical College, Hangzhou, Zhejiang 310014, China; Center for Plastic & Reconstructive Surgery, Department of Plastic & Reconstructive Surgery, Zhejiang Provincial People’s Hospital, Affiliated People’s Hospital, Hangzhou Medical College, Hangzhou, Zhejiang 310014, China; Department of Infectious Diseases, The Second Affiliated Hospital, Zhejiang University School of Medicine, Hangzhou, Zhejiang 310009, China; Center for Plastic & Reconstructive Surgery, Department of Plastic & Reconstructive Surgery, Zhejiang Provincial People’s Hospital, Affiliated People’s Hospital, Hangzhou Medical College, Hangzhou, Zhejiang 310014, China; Center for Plastic & Reconstructive Surgery, Department of Plastic & Reconstructive Surgery, Zhejiang Provincial People’s Hospital, Affiliated People’s Hospital, Hangzhou Medical College, Hangzhou, Zhejiang 310014, China; Center for Plastic & Reconstructive Surgery, Department of Plastic & Reconstructive Surgery, Zhejiang Provincial People’s Hospital, Affiliated People’s Hospital, Hangzhou Medical College, Hangzhou, Zhejiang 310014, China; Center for Plastic & Reconstructive Surgery, Department of Plastic & Reconstructive Surgery, Zhejiang Provincial People’s Hospital, Affiliated People’s Hospital, Hangzhou Medical College, Hangzhou, Zhejiang 310014, China; Center for Plastic & Reconstructive Surgery, Department of Plastic & Reconstructive Surgery, Zhejiang Provincial People’s Hospital, Affiliated People’s Hospital, Hangzhou Medical College, Hangzhou, Zhejiang 310014, China; Center for Plastic & Reconstructive Surgery, Department of Plastic & Reconstructive Surgery, Zhejiang Provincial People’s Hospital, Affiliated People’s Hospital, Hangzhou Medical College, Hangzhou, Zhejiang 310014, China

**Keywords:** ADSCs, conditioned media, gelma/algma (GA) hydrogels, chronic diabetic wounds

## Abstract

Chronic diabetic wounds remain a major clinical challenge owing to impaired angiogenesis, persistent inflammation and cellular dysfunction. In this study, we developed a bioadhesive composite hydrogel scaffold (gelatin methacryloyl/alginate methacrylate [GA]) fabricated using digital light processing three-dimensional (3D) bioprinting, in which human adipose-derived mesenchymal stem cells (ADSCs) or their conditioned medium (CM) was incorporated to generate 3D skin constructs, and compared the paracrine effects on skin regeneration. We characterized the microstructures of GA-ADSC and GA-CM scaffolds; profiled CM proteins and systematically compared the effects of GA-ADSCs and GA-CM on fibroblast proliferation, migration, angiogenesis and macrophage polarization *in vitro*. Their therapeutic efficacy was further evaluated in diabetic mouse wound models, including analyses of collagen deposition, angiogenesis and fibrosis markers. Furthermore, proteomic analysis was performed to understand the underlying mechanisms. *In vitro*, both GA-ADSCs and GA-CM promoted fibroblast proliferation, migration, angiogenesis and macrophage M2 polarization. Moreover, they accelerated wound closure in diabetic mice by enhancing collagen deposition and neovascularization (CD31) and suppressing α-smooth muscle actin and transforming growth factor-β1 expression. Notably, GA-ADSCs showed prolonged cell viability and exhibited stronger immunomodulatory and antifibrotic effects than GA-CM. Proteomic analysis revealed distinct mechanistic differences: GA-ADSCs markedly activated Toll-like receptor signaling, necroptosis and extracellular matrix remodeling pathways, suggesting their key roles in immune regulation and metabolic reprogramming. In contrast, GA-CM primarily activated the Hippo signaling pathway, consistent with its role in growth factor-mediated tissue repair. Furthermore, we compared the differentially regulated proteins between ADSCs and CM in diabetic wounds and showed that ADSCs significantly modulated RGL2, RSPH9 and CRTC3, which are associated with enhanced cellular function and energy metabolism. These findings provide mechanistic insights into ADSC- and CM-mediated effects and support the development of bioinspired stem cell-based therapeutic strategies for chronic diabetic wound repair.

## Graphical abstract

**Graphical Abstract:**
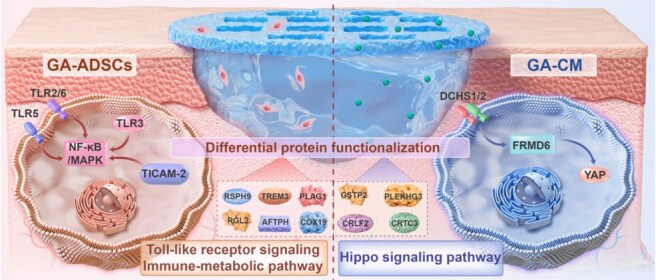

## Introduction

Chronic diabetic wounds (CDWs) are among the most common complications of diabetes, frequently leading to refractory ulcers, pathological scarring and lower limb amputations [1, 2]. Impaired angiogenesis, persistent inflammation and cellular dysfunction underlie the poor healing outcomes of CDWs, and conventional treatments remain insufficient, highlighting the urgent need for advanced regenerative strategies [3, 4]. Recently, mesenchymal stem cell-based therapies have emerged as a promising approach for skin tissue repair [5]. Human adipose-derived mesenchymal stem cells (ADSCs) are particularly attractive because of their abundance, ease of harvesting, immunocompatibility and potent paracrine activity [6–10]. However, their therapeutic efficacy is compromised by their poor survival and short-term retention in hostile wound microenvironment [11–13]. To overcome these limitations, cell-free approaches such as conditioned medium (CM) enriched with ADSC-derived secretomes have been explored as scalable and storable alternatives [14, 15]. Both ADSCs and CM have been reported to promote fibroblast proliferation, angiogenesis and macrophage polarization [11, 16–21]. Nevertheless, whether CM can fully substitute stem cell therapy in chronic wound repair and how their underlying regenerative mechanisms differ remain unclear.

Hydrogels offer versatile platforms for the stabilization and delivery of therapeutic cells or secretomes [22, 23]. Gelatin methacryloyl (GelMA) and alginate methacrylate (AlgMA) are particularly appealing because of their biocompatibility, tunable mechanics and intrinsic bioactivities. GelMA supports cell adhesion and differentiation, whereas AlgMA—widely used in wound dressings—provides structural stability and suppresses inflammatory cytokines [24, 25]. Previous studies using alginate–calcium ion crosslinking strategies have produced hydrogels with complex compositions and limited controllability. To address these limitations, GelMA and AlgMA can be combined (GelMA/AlgMA [GA]) via photocrosslinking to form an interpenetrating polymer network with enhanced integrity [26, 27]. Three-dimensional (3D) bioprinting enables precise spatial control of cells and materials, mimics native skin microarchitecture and improves graft integration [28].

In this study, we developed a bioadhesive GA hydrogel scaffold using digital light processing (DLP)-based 3D bioprinting for the delivery of ADSCs or CM to treat diabetic wounds. We characterized the microstructures of GA-ADSCs and GA-CM scaffolds; profiled CM proteins and systematically compared the effects of GA-ADSCs and GA-CM on fibroblast proliferation, migration, angiogenesis and macrophage polarization *in vitro*. Their therapeutic efficacy was further evaluated in diabetic mouse wound models, including analyses of collagen deposition, angiogenesis (CD31) and fibrosis markers (α-smooth muscle actin [α-SMA] and transforming growth factor-β1 [TGF-β1]). Live imaging was used to track the survival and retention of ADSCs *in vivo*. Importantly, proteomic analyses revealed distinct mechanisms: ADSCs primarily activated Toll-like receptor (TLR) signaling associated with immune regulation, resolution of chronic inflammation and antifibrotic remodeling, whereas CM predominantly engaged in Hippo signaling, consistent with its role in growth factor-driven cell proliferation and tissue repair. Differentially regulated proteins such as RGL2, RSPH9 and CRTC3 were also identified, indicating their roles in cellular function and energy metabolism in refractory wound repair.

Collectively, this study provides new mechanistic insights into cell-based vs. cell-free therapies and shows that GA hydrogel scaffolds as multifunctional 3D-bioprinted platforms with strong translational potential for the treatment of CDWs.

## Materials and methods

### Reagents and materials

Cell culture media, supplements, antibodies and hydrogel components were purchased from the indicated suppliers. Key reagents included DMEM/high glucose, α-MEM, FBS (Gibco), GelMA and AlgMA (EFL-Gem), LAP photoinitiator, antibodies for CD29, CD44, CD73, CD90, CD105, CD34, CD45, CD80, CD206 and dyes including Calcein-AM, PI, DAPI, DiD and Crystal Violet.

### Preparation of GelMA/AlgMA hydrogels

GelMA and AlgMA were dissolved in LAP photoinitiator solution, filtered and stored at 4°C in the dark. Hydrogels were photopolymerized under 365 nm ultraviolet (UV) light, and their macroscopic gelation and pore structures were characterized by scanning electron microscopy (SEM). Fourier Transform Infrared (FTIR) spectroscopy confirmed chemical structures. Swelling and enzymatic degradation were evaluated in phosphate-buffered saline (PBS) at 37°C, and rheological properties were measured using a rotational rheometer.

### Isolation and characterization of hADSCs

Human adipose tissue from donors (<40 years) was digested with collagenase, filtered and cultured in α-MEM supplemented with 10% FBS. MSC identity was confirmed by flow cytometry (CD29^+^, CD44^+^, CD73^+^, CD90^+^, CD105^+^; CD34^−^, CD45^−^) and multilineage differentiation assays (adipogenic and osteogenic). Passage 3 hADSCs were encapsulated in GelMA/AlgMA hydrogels (1 × 10^7^ cells/mL) and photopolymerized via DLP 3D printing.

### Extraction of human ADSCs CM

ADSCs within passage 20 (P20) were seeded into 10 cm culture dishes and cultured until the cell confluence reached approximately 90%, at which point the cell number was about 15 × 10^5^. The original complete medium was discarded, and the cells were gently washed 2–3 times with sterile PBS to thoroughly remove residual serum and metabolic debris. Subsequently, the medium was replaced with serum-free basic medium, and the cells were statically cultured in an incubator at 37°C with 5% CO_2_ for 48 h.

After the culture period, the supernatant was collected under sterile conditions. First, the supernatant was centrifuged at 300*g* for 10 min to remove viable cells, then centrifuged at 2000*g* for 10 min to remove cell debris. The supernatant was collected and filtered through a 0.22 μm filter membrane for sterilization to obtain the primary CM. Subsequently, a lyophilizer was used to lyophilize and concentrate the CM, and finally, high-activity lyophilized powder of ADSCs CM was obtained. The powder was aliquoted and stored at −80°C for later use. For subsequent experiments, it could be prepared into the corresponding concentration using PBS solution.

### *In vitro* cytocompatibility and functional assays

Human Dermal Fibroblasts (HDFs) were co-cultured with different hydrogel formulations (control, GA, CM, ADSCs, GA-CM, GA-ADSCs) in transwell chambers. Cell viability, proliferation and migration were assessed using live/dead staining, CCK-8 assay and scratch assays. Endothelial tube formation was performed on Matrigel-coated plates using HUVECs, and tube networks were quantified.

### Hemolysis assay

Hydrogels were incubated with human erythrocytes to evaluate hemolytic activity. PBS and deionized water served as negative and positive controls. Absorbance at 545  nm was used to calculate hemolysis rate.

### *In vivo* diabetic wound model

ICR mice (male, 4–5-week-old) and BALB/c-nu mice (male, 4–5-week-old) were procured from Hangzhou Qizhen Experimental Animal Technology Co., Ltd. (Hangzhou, China). All animal experiments were approved by the Animal Ethics Committee of Zhejiang Provincial People’s Hospital and were conducted in accordance with the ARRIVE guidelines 2.0. Mice were maintained under specific-pathogen-free (SPF) conditions (22 ± 2°C, 50 ± 10% humidity, 12-h light/dark cycle) and acclimatized for 1 week before experiments. All animals were genetically unmodified and had no prior procedures.

To induce diabetes, mice were intraperitoneally injected with STZ (50 mg/kg) for five consecutive days. For surgical procedures, mice were anesthetized with isoflurane. Full-thickness circular dorsal wounds (10 mm) were created and treated with PBS, hADSCs, CM, GA, GA-ADSCs or GA-CM, with six mice per group. Animals were excluded from this study if they died before the conclusion of the experiment; however, no samples were excluded during the data analysis. Wound healing was documented on days 1, 3, 7, 10 and 14. All outcome assessments were conducted by experimenters who were blinded to the group assignments. Tissues were collected for histological, immunofluorescent and molecular analyses. At the end of the experiments, mice were euthanized by intraperitoneal injection of sodium pentobarbital (200 mg/kg). Following administration, animals were carefully monitored until complete cessation of respiration and heartbeat, with a secondary physical method applied when necessary to confirm death.

### Histological and immunohistochemical analyses

On day 14 after wound creation, the dorsal full-thickness skin wounds of mice were excised. Each wound was bisected longitudinally: one half was fixed in 4% paraformaldehyde for histological and immunofluorescence analyses, and the other half was immediately stored at −80°C for subsequent proteomic analysis.

For histological evaluation, fixed tissues were dehydrated through graded ethanol, embedded in paraffin and cut into 4 µm-thick sections. Sections were stained with hematoxylin and eosin (H&E) to assess general tissue morphology and with Masson’s trichrome to evaluate collagen deposition. For each wound, at least three randomly selected sections were analyzed in a blinded manner.

For immunofluorescence analysis, paraffin sections were deparaffinized, rehydrated and subjected to antigen retrieval. Sections were then blocked with 5% bovine serum albumin and incubated overnight at 4°C with primary antibodies targeting markers of angiogenesis (CD31), TGF-β1 and macrophage polarization (CD80, CD206). After washing, sections were incubated with appropriate fluorescently labeled secondary antibodies. Nuclei were counterstained with DAPI, and sections were imaged using a fluorescence microscope.

### Western blot analysis

Total cellular proteins of HDFs were extracted with RIPA buffer containing proteinase inhibitor cocktail for 30 min on ice. The targeted protein was separated by denaturing sodium dodecyl sulfate polyacrylamide gel electrophoresis (8–12%) and transferred onto a nitrocellulose membrane. The membrane was blocked with 5% nonfat milk for 2 h, which was followed by overnight incubation at 4°C with the following primary antibodies against β-actin, NF-κB and IL-6. Following incubation with peroxidase-conjugated goat anti-rabbit/mouse IgG at 4°C for 2 h, each protein was visualized using Western Lightning^®^ Plus ECL, detected using X-ray film and scanned. Each experiment was conducted in triplicate.

### Real time PCR

The mRNA expression of targeted genes in HDFs cells was measured using a qPCR assay on an ABI QuantStudio™ 7 Flex Real-Time PCR System (Applied Biosystems; Thermo Scientific, USA). Total RNA was extracted with TRIzol reagent and quality controlled by NanoDrop2000 spectrophotometer (Thermo Scientific, USA). cDNA reverse transcription was performed by using All-in-One cDNA Synthesis SuperMix. The PCR system was 20 μL, including 10 μL SYBR Green qPCR Master Mix (low ROX), 0.4 μL PCR forward primer, 0.4 μl PCR reverse primer, 1 μL template cDNA and 8.2 μL ddH2O, with the following reaction conditions: initial denaturation at 95°C for 5 min, 40 cycles of denaturation at 95°C for 3 s and annealing and extension at 60°C for 30 s. β-Actin was used as reference gene and the 2^−ΔΔCT^ method was used to analyze the relative mRNA expressions (Table 1). Each experiment was conducted in triplicate.

**Table 1:** Primer sequences used for qPCR analysis.

Gene	Forward primer	Reverse primer
*GAPDH*	5′-*AGATCCCTCCAAAATCAAGTGG-3′*	5′-*GGCAGAGATGATGACCCTTTT*-3′
*YAP1*	5′-TGTCCCAGATGAACGTCACAGC-3′	5′-TGGTGGCTGTTTCACTGGAGCA-3′
*CTGF*	5′-*CAGCATGGACGTTCGTCTG*-3′	5′-*AACCACGGTTTGGTCCTTGG*-3′
*TLR3*	5′-*TCAACTCAGAAGATTACCAGCCG*-3′	5′-*AGTTCAGTCAAATTCGTGCAGAA*-3′
*CXCL8*	*Beyotime: QH03705S*	
*RGL2*	*Beyotime: QH21245S*	
*CRTC3*	5′-*CCCATACACGCTGCTGATGAT*-3′	5′-*GGAGGTTTCGTACCAGTAGTCA*-3′

### Data analysis

All experiments were performed in triplicate. Data are presented as mean ± SD. Statistical analyses were conducted using one-way ANOVA with Tukey’s *post hoc* test. Predefined criteria were applied for data point exclusion: outliers >3 standard deviations from the group mean (Grubbs’ test, *α* = 0.05) and incomplete or invalid measurements (e.g. equipment malfunction) were removed from the analysis. *P*-values were denoted as ns (*P* > 0.05), * (*P* ≤ 0.05), ** (*P* ≤ 0.01), *** (*P* ≤ 0.001) and **** (*P* ≤ 0.0001).

## Results

### Structural and physicochemical properties of 3D-printed GA hydrogels

**Figure 1:**
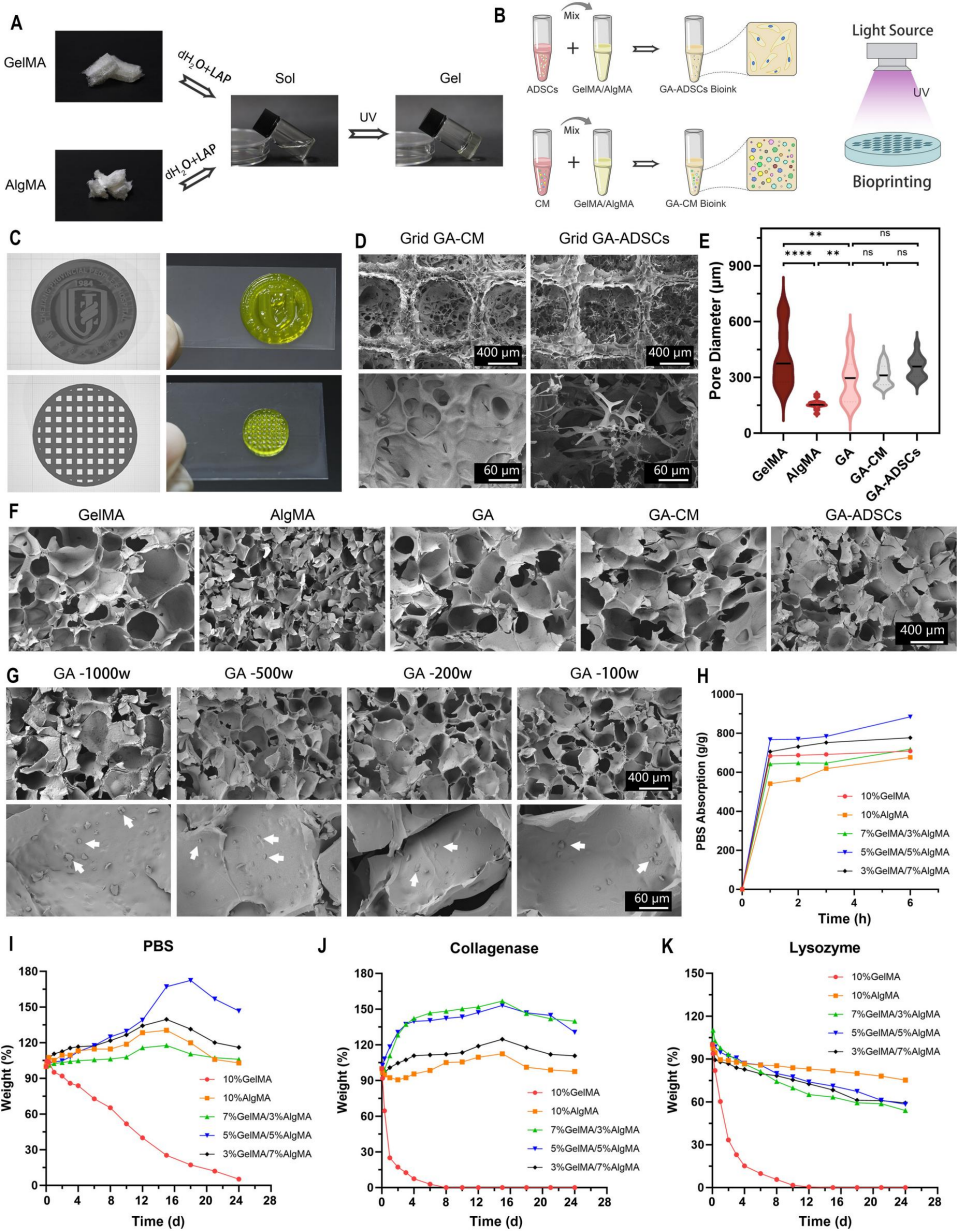
(**A**) Representative photographs of GelMA and AlgMA before and after mixing with LAP and their sol–gel transition under UV irradiation. (**B**) Schematic diagram of GA bioink preparation and DLP 3D printing of porous patches. (**C**) Photographs of 3D-printed GA hydrogel patches show the hospital emblem and grid structures. (**D**) SEM images of GA-CM and GA-ADSCs grid patches after 3 days of culture. (**E**) Quantitative analysis of the pore diameters in GelMA, AlgMA, GA, GA-CM and GA-ADSCs. (**F**) SEM images of freeze-dried GelMA, AlgMA, GA, GA-CM and GA-ADSCs hydrogels. (**G**) SEM images of GA hydrogels seeded with different initial cell densities after 3 days of culture show encapsulated cells adhering to the pore walls. (**H**) Swelling ratios of freeze-dried hydrogels in PBS at 37°C. Degradation profiles of non-freeze-dried hydrogels in (**I**) PBS, (**J**) collagenase solution and (**K**) lysozyme solution.

To construct hydrogel scaffolds with favorable cell-loading capacity and structural stability, a GA composite hydrogel was fabricated using DLP 3D printing. In this context, this study employed GelMA and AlgMA as raw materials, both of which contain methacryloyl groups capable of photocrosslinking, enabling rapid photopolymerization under UV light. Under UV irradiation in the presence of a light-activated polymer, GelMA and AlgMA underwent rapid photocrosslinking (Figure 1A, Supplementary Figure S1). The fabrication workflow and printing of GA-CM and GA-ADSCs are shown in Figure 1B and C, and complex patterns were faithfully reproduced with a stable morphology. SEM revealed distinct pore sizes in the single-component hydrogels, with GelMA exhibiting the largest pores and AlgMA exhibiting the smallest pores. The GA composite hydrogel displayed a homogeneous porous structure, which was preserved after loading with CM or ADSCs (Figure 1F). Quantitative pore size analysis showed that GelMA had the largest pore diameter (410.3 ± 154.0 µm), whereas AlgMA had the smallest pore diameter (155.9 ± 25.08 µm). GA, GA-CM and GA-ADSCs displayed no significant differences (Figure 1E). Tukey’s multiple comparison analysis further confirmed the absence of statistical differences between GA and its two loading forms (GA-CM and GA-ADSCs), indicating that the blending and loading processes did not significantly affect the structural stability of the scaffolds.

After 3 days of culture, SEM observations revealed that cells in the GA-ADSCs group adhered along the pores and formed interlaced arrangements, whereas no obvious cellular morphology was observed on the surface of the GA-CM group (Figure 1D). Figure 1G shows the effects of different initial cell densities (1 × 10^6^–1 × 10^7^ cells/mL) on embedding efficiency within the scaffolds, with cells observed along the inner surfaces and edges of the hydrogel pores, suggesting that GA scaffolds possess excellent cell encapsulation and adhesion properties.

We further evaluated the swelling and degradation properties of hydrogels with different GelMA/AlgMA composition ratios to assess their water retention capacity and metabolic safety *in vivo*. Six concentration gradient groups were designed in the experiment, namely: 10% GelMA group, 10% AlgMA group, 7% GelMA/3% AlgMA group, 5% GelMA/5% AlgMA group, 3% GelMA/7% AlgMA group, to explore the influence of different component ratios on the properties of hydrogels. Upon immersion of freeze-dried hydrogels in PBS at 37°C, all the groups exhibited rapid swelling within the first hour, followed by a slow increase thereafter (Figure 1H). At 6 h, the swelling ratio differed significantly among formulations: the 5% GelMA/5% AlgMA group reached the highest value (884.2 ± 84.76%), whereas the 10% AlgMA group displayed the lowest value (676.7 ± 73.45%), indicating that GA composites promoted water uptake more efficiently than AlgMA alone (Supplementary Figure S2). In degradation assays using non-freeze-dried samples (Figure 1I–K), distinct composition-dependent behaviors were observed. In PBS, the 10% GelMA group degraded rapidly and was nearly lost by day 24, whereas 10% AlgMA maintained approximately 90–100% of its weight with minimal change. The GA composites (7/3, 5/5 and 3/7) exhibited moderate swelling (peaking at approximately 120–170%), followed by a gradual decline; however, they retained >100% of their initial weight at later stages. In the collagenase solution, 10% GelMA was completely degraded within 4 days, whereas 10% AlgMA remained highly stable. The 7% GelMA/3% AlgMA composite exhibited strong and sustained resistance to collagenase, maintaining 120–140% of its mass for more than 2 weeks before gradually decreasing, indicating that AlgMA markedly protected GelMA from enzymatic degradation. In the lysozyme environment, 10% GelMA was almost completely degraded within 7 days, whereas 10% AlgMA degraded slowly, retaining approximately 80–90% of its mass on day 24. The 7% GelMA/3% AlgMA composite showed a gradual decrease over time, but its structural integrity was preserved throughout the 24-day period. Collectively, these results indicate that AlgMA alone exhibits lower swelling capacity but higher enzymatic stability than GelMA and that the incorporation of AlgMA into GelMA networks significantly enhances long-term structural stability.

Rheological analysis under UV irradiation (365 nm, 90 W) revealed rapid gelation across all formulations, with a sharp increase in the storage modulus (*G*′) after approximately 10 s of light exposure (Supplementary Figure S3). In all groups, *G*′ surpassed *G*″ within 30–40 s, indicating stable crosslinking and tunable stiffness depending on the GelMA/AlgMA ratio. Higher AlgMA fractions slightly increased the final modulus, suggesting a reinforcing effect of ionic interactions. FTIR spectra further confirmed successful hydrogel formation (Supplementary Figure S4), showing characteristic peaks of –OH/N–H (∼3430–3440 cm^−1^), C–H (∼2927–2928 cm^−1^), amide I/II (1650–1547 cm^−1^) and C–O–C/C–O (∼1246–1030 cm^−1^), indicating the coexistence of hydrogen bonding, ionic coordination and covalent methacrylate crosslinking. These results demonstrate that the GA system integrates ionic and covalent crosslinking, contributing to improved swelling–degradation balance, rapid photocuring and enhanced mechanical stability [29].

In conclusion, based on porosity, swelling, degradation and mechanical evaluations, the optimal GelMA/AlgMA ratio was determined to be 7:3. A slightly higher content of GelMA than AlgMA can not only ensure the flexibility and mechanical stability of the hydrogel, but also maintain the stability of the water absorption and degradation rate of the hydrogel.

### Characterization of ADSCs, paracrine profiles of ADSC-CM and biocompatibility of 3D-printed GA hydrogels

ADSCs were successfully isolated via collagenase digestion and exhibited a typical fibroblast-like morphology under phase-contrast microscopy (Supplementary Figure S5). At 80–90% confluence, the cells were aligned in spiral clusters, indicating strong proliferative capacity. Multilineage differentiation was confirmed using Oil Red O and Alizarin Red S staining, which demonstrated lipid droplet formation and calcified nodule deposition after adipogenic and osteogenic induction, respectively (Supplementary Figure S6). Flow cytometry further verified the high expression of CD29, CD44, CD73, CD90 and CD105 and the absence of the hematopoietic markers CD34 and CD45, consistent with the International Society for Cell & Gene Therapy criteria for mesenchymal stem cells (Supplementary Figure S7).

BCA concentration detection was performed on the CM produced by culturing ADSCs (approximately 15 × 10^5^ cells) for 48 h (i.e. CM group) and the medium without cell treatment (i.e. control group) (Supplementary Figure S8). The results showed that the control group contained a protein content of 2.4470 ± 0.077 mg, while the CM group contained a protein content of 2.5098 ± 0.116 mg, with a difference of approximately 0.0628 mg between the two groups. After lyophilization and concentration of the conditioned media of the control group and CM group, 1 mL of PBS buffer was added to the powder for BCA concentration detection. The detection results showed that the control group contained a protein content of 2.1234 ± 0.076 mg, and the CM group contained a protein content of 2.1857 ± 0.088 mg, with a difference of approximately 0.0623 mg between the two groups.

**Figure 2:**
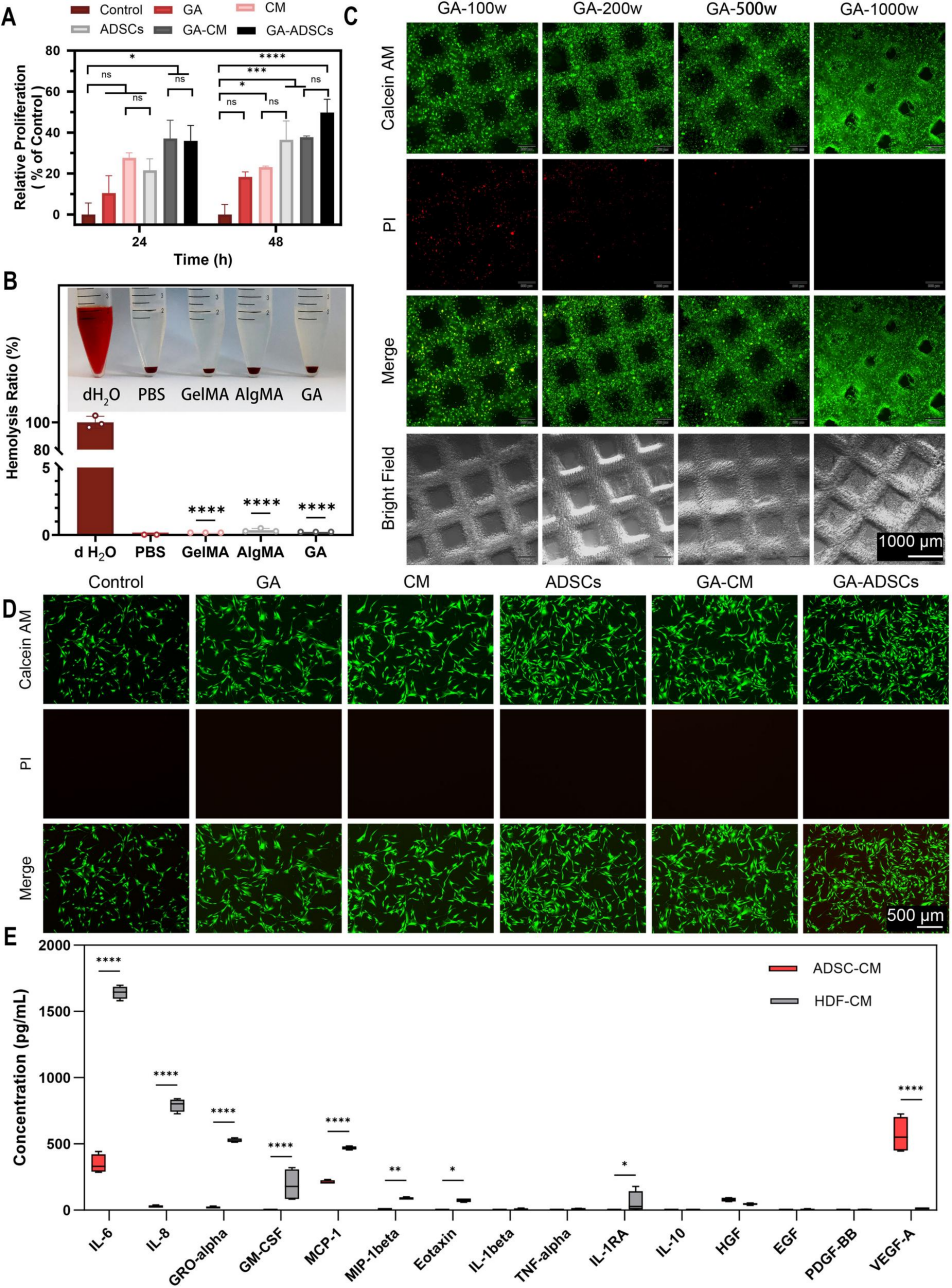
(**A**) Proliferation of HDFs cultured with different treatments, as determined using CCK-8 assay at 24 and 48 h. (**B**) Hemolysis ratio of hydrogels compared with positive (dH_2_O) and negative (PBS) controls. (**C**) Live/dead staining of ADSCs seeded on GA scaffolds at different initial densities. (**D**) Live/dead staining of HDFs in different treatment groups, including control, GA, CM, ADSCs, GA-CM and GA-ADSCs. (**E**) Cytokine profiling of ADSCs-CM and HDFs-CM shows distinct paracrine features.

The biocompatibility of GA hydrogels was systematically assessed. Cell Counting Kit-8 assays revealed that GA-CM and GA-ADSCs significantly enhanced human dermal fibroblast (HDF) proliferation at 24 h compared with the control, with GA-ADSCs showing the strongest effect at 48 h (Figure 2A). Hemolysis assays confirmed excellent hemocompatibility, with GelMA, AlgMA and GA hydrogels exhibiting negligible hemolytic activity, similar to that of PBS (Figure 2B). The extremely low hemolysis rate of GA hydrogels indicates that they do not cause obvious damage to red blood cells, which is a key prerequisite for their *in vivo* application and further confirms their good biocompatibility from the perspective of hemocompatibility. Live/dead staining of ADSCs encapsulated in GA scaffolds at different seeding densities (1 × 10^6^–1 × 10^7^ cells/mL) showed high viability after 7 days, reaching 99.42 ± 0.20% in the 1 × 10^7^ group and remaining at 91.48 ± 1.82% even at the lowest density (Figure 2C, Supplementary Figure S9). Comparative live/dead staining of HDFs co-cultured with different hydrogel groups (control, GA, CM, ADSCs, GA-CM and GA-ADSCs) confirmed excellent cytocompatibility, with quantified cell survival rates exceeding 99.9% in all groups (Figure 2D, Supplementary Figure S10).

Cytokine profiling further revealed distinct paracrine signatures between ADSC-CM and HDF-CM. ADSC-CM was enriched in vascular endothelial growth factor (VEGF) A, a key pro-angiogenic factor, whereas HDF-CM contained higher levels of pro-inflammatory cytokines such as interleukin-6 (IL-6), interleukin-8 (IL-8), granulocyte-macrophage colony-stimulating factor (GM-CSF) and monocyte chemoattractant protein 1 (MCP-1). Interleukin-1 receptor antagonist (IL-1Ra) was slightly elevated in HDF-CM (*P *< 0.05), whereas interleukin-10 (IL-10), hepatocyte growth factor, epidermal growth factor (EGF) and platelet-derived growth factor-BB (PDGF-BB) showed no significant differences (Figure 2E), indicating that ADSC-CM exerts its regenerative potential by providing anti-inflammatory and pro-angiogenic factors.

Taken together, these findings confirm the successful isolation and characterization of ADSCs, demonstrate that ADSCs can secrete and accumulate a certain amount of soluble proteins during 48-h culture (as evidenced by BCA detection results showing that the protein content of CM group is slightly higher than that of the control group, and the relative difference remains stable after lyophilization and concentration, Supplementary Figure S8), highlight the paracrine advantages of ADSC-CM and demonstrate that DLP 3D-printed GA hydrogels provide a structurally stable and highly biocompatible microenvironment. The scaffolds not only supported fibroblast proliferation and ADSCs survival but also enhanced cellular activity when combined with ADSCs or their CM, underscoring the advantages of integrating 3D bioprinting with cell- and secretome-based therapies for chronic wound repair.

### *In vitro* effects of GA-ADSCs and GA-CM on key skin-associated cells

**Figure 3:**
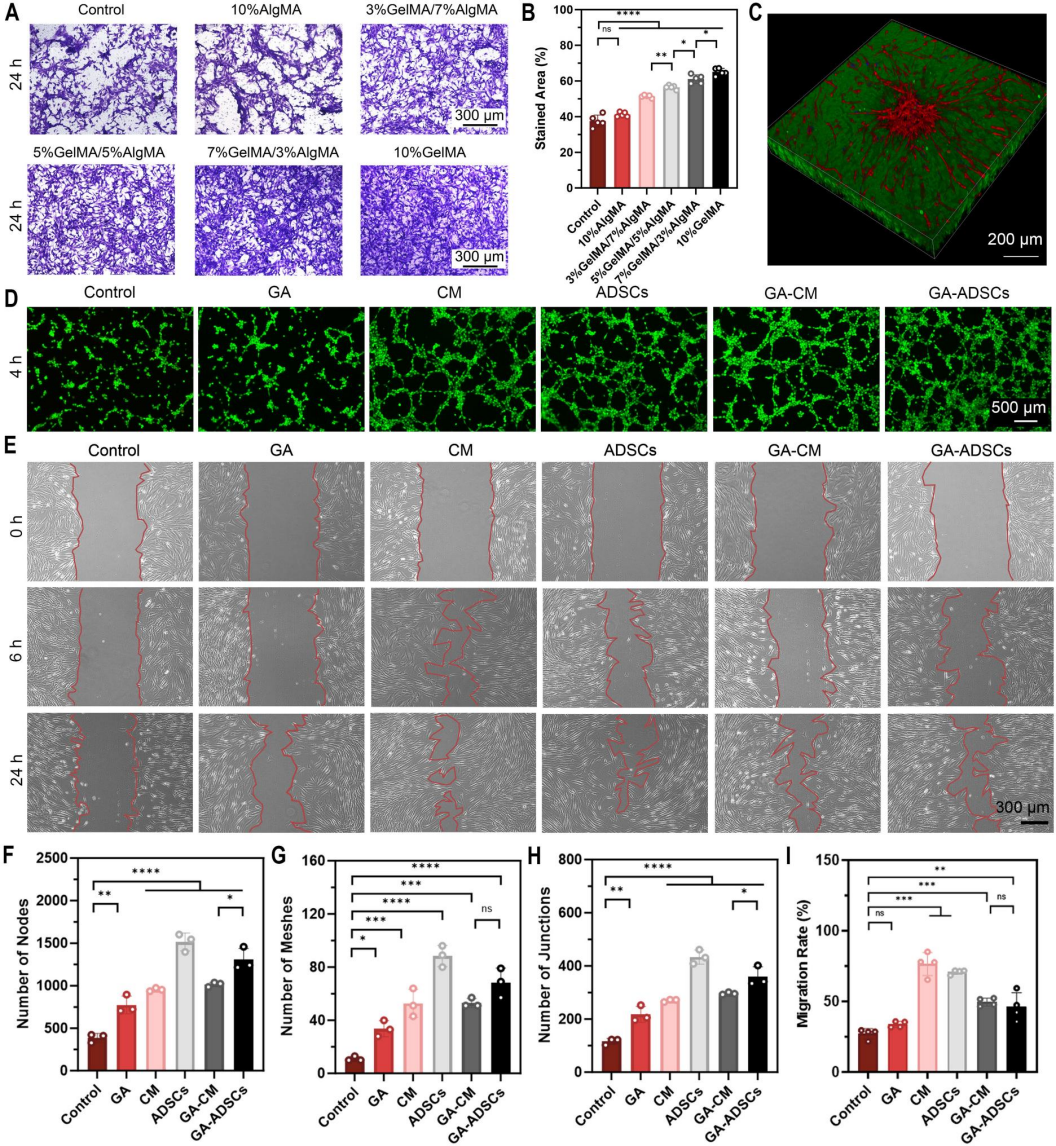
(**A**) Transwell migration assay of human ADSCs in response to different GelMA/AlgMA hydrogel formulations. (B) Quantitative analysis of stained areas shows enhanced migration in the 5% GelMA/5% AlgMA, 7% GelMA/3% AlgMA and 10% GelMA groups compared with the control group. (**C**) 3D confocal imaging of ADSCs in GA hydrogel. ADSCs were stained with phalloidin and GA hydrogel with fluorescent dye. (**D**) Tube formation assay shows vascular-like network formation in the control, GA, CM, ADSCs, GA-CM and GA-ADSCs groups. (**E**) Scratch wound assay of HDFs at 0, 6 and 24 h under different treatments. Quantitative analysis of tube formation, including number of (F) nodes, (**G**) meshes and (H) junctions. (**I**) Quantitative analysis of the fibroblast migration rate in scratch assay.

To investigate the pro-migratory, proliferative, pro-angiogenic and anti-inflammatory effects of GA-ADSCs and GA-CM, we systematically assessed their effects on cell migration, angiogenesis and macrophage polarization *in vitro*. To investigate the functional effects of different hydrogel compositions, a Transwell migration assay was performed using ADSCs. Hydrogels with moderate-to-high GelMA content (5% GelMA/5% AlgMA, 7% GelMA/3% AlgMA and 10% GelMA) showed markedly enhanced cell migration compared with the control and 10% AlgMA groups (*P* < 0.0001, Figure 3A and B). No significant difference was observed between 7% GelMA/3% AlgMA and 10% GelMA, and both outperformed 3% GelMA/7% AlgMA. Confocal 3D imaging showed that ADSCs adhered well to the hydrogel surface and spread fully, with more cells aggregated in the pores of the hydrogel (Figure 3C). Based on the above results, this study further confirms that the 7% GelMA/3% AlgMA ratio is more suitable for subsequent experimental research.

Subsequently, angiogenic potential was assessed using a tube formation assay with human umbilical vein endothelial cells (Figure 3D). Compared with the control group, both CM and ADSCs markedly enhanced vascular-like network formation, with ADSCs exhibiting stronger pro-angiogenic activity than CM (1514.00 ± 105.30 nodes, 433.30 ± 26.58 junctions, 88.33 ± 8.02 meshes, *P* < 0.0001). Importantly, GA-CM and GA-ADSCs significantly promoted angiogenesis through sustained release within the 3D scaffolds, showing clear improvements over GA alone, with GA-ADSCs outperforming GA-CM (Figure 3F–H; *P* < 0.01). Scratch assays further confirmed the pro-migratory effects of GA-ADSCs and GA-CM. As shown in Figure 3E, within 24 h, the migration rates (the blank ratios of wound area) of CM and ADSCs reached 76.50 ± 8.18% and 70.85 ± 1.65%, respectively, both significantly higher than that of the control group (*P* < 0.0001). GA-CM and GA-ADSCs also promoted fibroblast migration, with no statistically significant differences between the two groups (Figure 3I).

**Figure 4:**
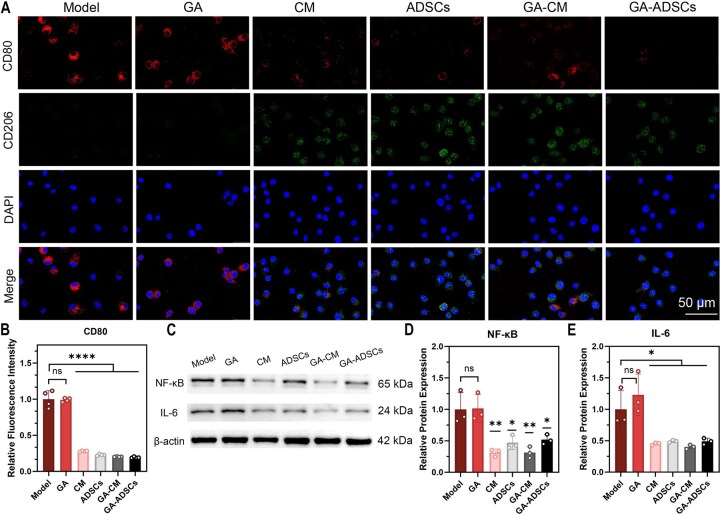
(**A**) Immunofluorescence staining of RAW 264.7 cells for the M1 marker CD80 and M2 marker CD206. (**B**) Analysis of relative fluorescence intensity of immunofluorescence, with the fluorescence intensity of model group set as 1 for control. (**C**) WB results of RAW 264.7 cells, including indicators of NF-κB, IL-6 and internal reference β-actin. (**D** and **E**) Quantitative analysis of relative NF-κB and IL-6 protein expression by WB, with the protein expression of model group set as for control.

Finally, the immunomodulatory ability and anti-inflammatory effect of the samples were evaluated using RAW 264.7 macrophages stimulated by lipopolysaccharide (LPS) combined with interferon‑gamma (IFN-γ) (Figure 4A). LPS combined with IFN-γ alone induced M1 polarization of macrophages, while treatment with ADSCs, GA-ADSCs, CM and GA-CM significantly promoted the polarization of macrophages to the M2 phenotype. Specifically, the immunofluorescence intensity of CD80 (a marker of M1 phenotype) in RAW 264.7 cells was significantly reduced in the active ingredient groups (ADSCs, GA-ADSCs, CM, GA-CM) compared with the model group (*P* < 0.0001), indicating that both ADSCs and CM could inhibit the M1 polarization of macrophages. There was no significant difference in the fluorescence intensity of CD80 among the ADSCs, GA-ADSCs, CM and GA-CM (*P* > 0.*05*), suggesting that the presence of GA did not affect the anti-inflammatory effect of the active ingredients (Figure 4B).

In addition, Western blot (WB) analysis was performed on RAW 264.7 cells using the same modeling method to detect the expression levels of NF-κB and IL-6 (Figure 4C and D). The results showed that, compared with the model group, the expression levels of NF-κB and IL-6 in all the active component groups (ADSCs, GA-ADSCs, CM, GA-CM) were significantly decreased (*P* < 0.05), further confirming the anti-inflammatory activity of ADSCs and CM.

In summary, all groups containing active ingredients (ADSCs, GA-ADSCs, CM, GA-CM) could exert effects in promoting cell migration, angiogenesis, anti-inflammation and immune regulation. In the *in vitro* cell experiments, the groups containing CM components (CM, GA-CM) showed slightly better effects than the groups containing ADSCs components (ADSCs, GA-ADSCs). This may be because CM can quickly release various effective components (such as cytokines and vesicles), while the role of ADSCs depends on the accumulation of time. In addition, the effect of the GA-wrapped groups (GA-ADSCs, GA-CM) was slightly better than that of the uncoated groups (ADSCs, CM), which may be related to the slow-release effect of GA, which can prolong the action time of active ingredients. However, the *in vitro* cell experiment has limitations in the action cycle and detection indicators, so further verification in animal experiments is needed to confirm the long-term therapeutic effect and mechanism of action of each group, so as to provide a more sufficient experimental basis for clinical transformation.

### *In vivo* effects of GA-ADSCs and GA-CM on wound healing of mice

**Figure 5:**
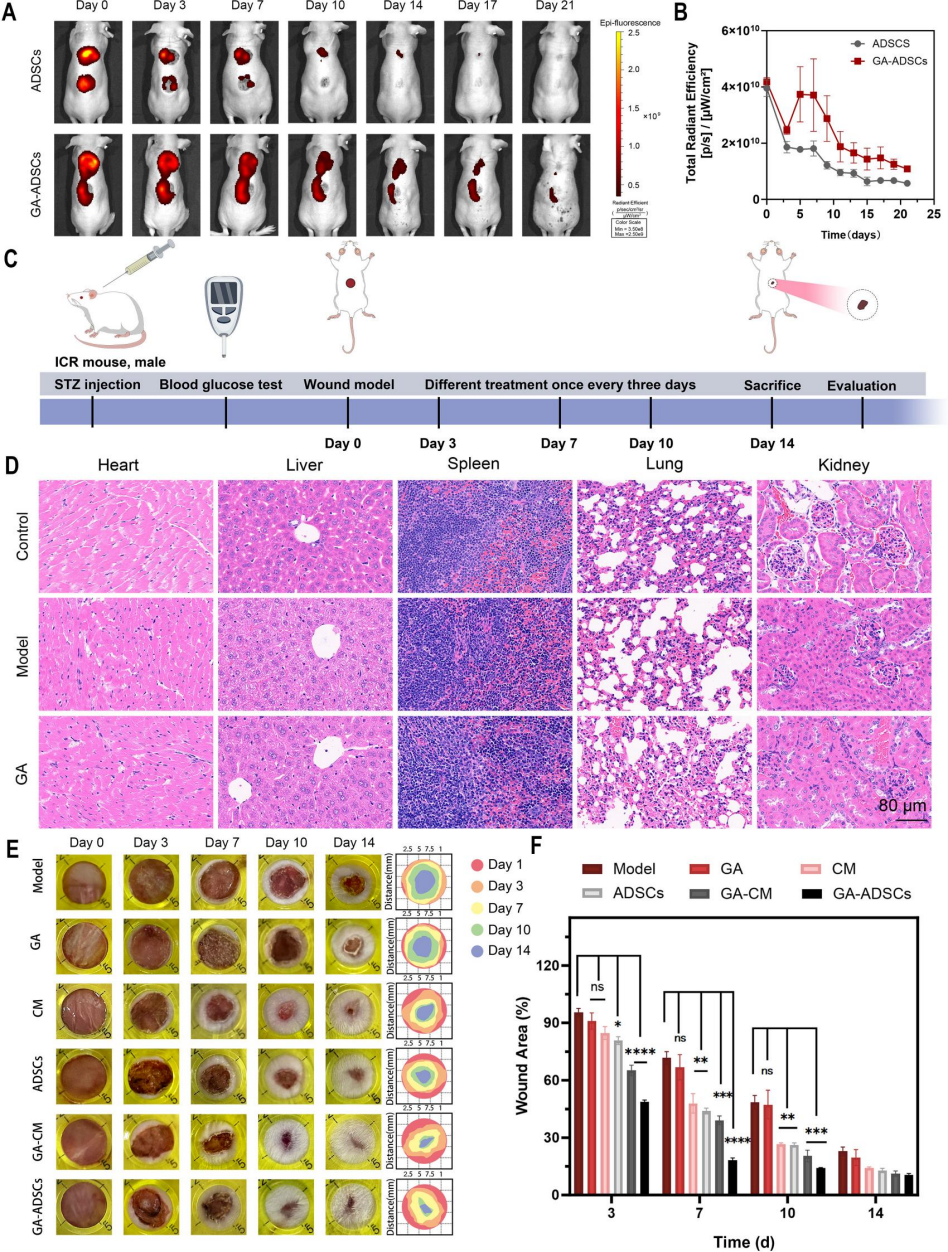
(**A**) In vivo tracking of DiD-labeled ADSCs show prolonged retention in the GA-ADSC group compared with ADSCs alone. (**B**) Quantitative fluorescence intensity analysis of ADSCs in vivo. (**C**) Schematic diagram of the diabetic wound model establishment and treatment protocol. (**D**) H&E of heart, liver, spleen, lung and kidney tissues in normal ICR mice, diabetic ICR mice and diabetic ICR mice following GA treatment. (**E**) Representative wound images from different treatment groups on days 0, 3, 7, 10 and 14. (**F**) Quantitative analysis of wound closure rates over time.

*In vivo* tracking of 1,1′-dioctadecyl-3,3,3′,3′-tetramethylindodicarbocyanine perchlorate-labeled ADSCs revealed that GA-ADSCs maintained strong fluorescence signals at the wound sites up to day 21, whereas free ADSCs rapidly declined by day 7 (Figure 5A and B), indicating that GA hydrogels significantly prolonged retention of ADSCs cells. A diabetic mouse model was successfully established via streptozotocin injection and was validated via blood glucose monitoring and histological examination, followed by the creation of full-thickness dorsal wounds (Figure 5C). H&E staining in Figure 5D showed that the heart, liver, spleen, lung and kidney of diabetic mice all exhibited typical pathological changes of multi-organ damage in type 2 diabetes, further confirming the successful establishment of the model. After applying GA hydrogel to the wound and continuous intervention for 14 days, H&E staining results showed that it did not cause obvious pathological damage or malignant changes in various organs, indicating that it has good *in vivo* biocompatibility.

As shown in Figure 5E–F, wound closure was the slowest in the model and GA groups, with large unhealed areas still evident by day 14. On day 10, wound areas remained at 48.54 ± 6.10% in the model group and 47.25 ± 13.05% in the GA group, with no significant difference (*P *> 0.05). In contrast, the CM and ADSC groups showed accelerated wound closure from day 7 and near-complete healing by day 14. Notably, GA-CM and GA-ADSCs exhibited the most pronounced therapeutic effects, with wound areas reduced to 17.09 ± 2.36% and 14.07 ± 0.54% on day 10, respectively (*P* < 0.001), and nearly complete closure by day 14. GA-ADSCs showed better wound healing with smaller residual scars than GA-CM, thereby outperforming GA-CM. Collectively, these results demonstrate that GA scaffolds markedly enhanced ADSC retention and therapeutic efficacy, with GA-ADSCs achieving the most effective wound healing in the diabetic mouse model.

### Histopathological and immunofluorescence analysis of wound healing in diabetic mice with GA-ADSCs and GA-CM treatment

**Figure 6:**
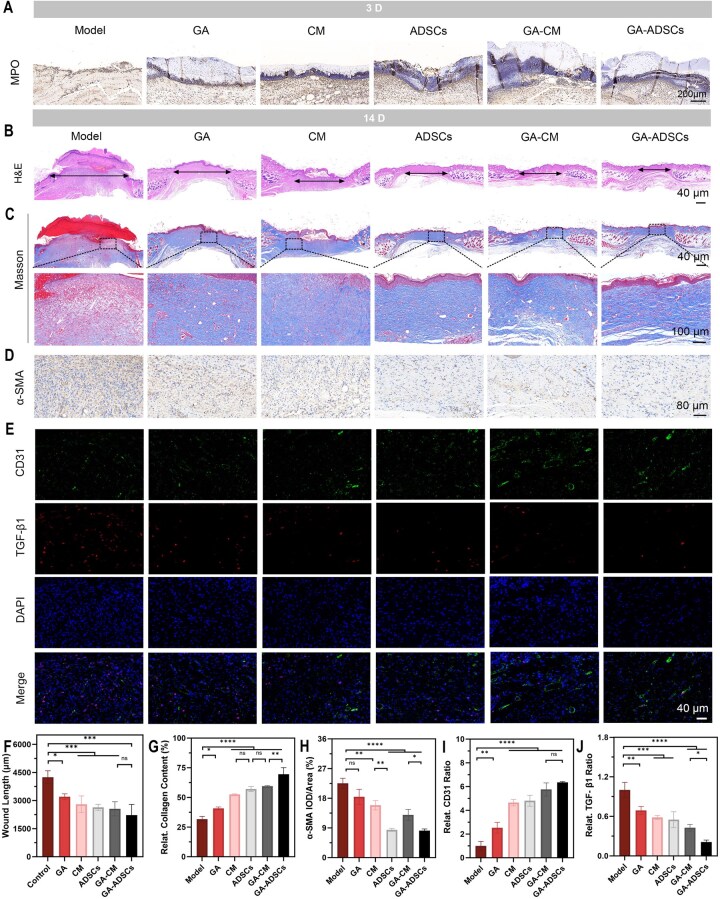
(**A**) MPO immunohistochemical staining of wound tissues on day 3. (**B**) Histological observation using H&E staining of wound edge closure on day 14. (**C**) Masson’s trichrome staining shows collagen deposition on day 14. (D) α-SMA immunohistochemical staining shows myofibroblast activation on day 14. (**E**) Immunofluorescence staining for CD31 and TGF-β1 on day 14. Quantitative analysis of (**F**) wound length, (**G**) relative collagen content, (**H**) α-SMA IOD/area ratio, (**I**) CD31 expression ratio and (**J**) TGF-β1 expression ratio (*n* ≥ 3).

Histological and immunofluorescence analyses were performed to evaluate collagen remodeling, myofibroblast activation and angiogenesis in diabetic wounds. On day 3, myeloperoxidase (MPO) immunohistochemistry revealed sparse neutrophil infiltration in the model group, indicating delayed inflammation, whereas CM, ADSCs, GA-CM and GA-ADSCs markedly increased the number of MPO-positive cells, suggesting that these treatments facilitated a rapid transition into the early inflammatory immune phase (Figure 6A). Notably, the GA group also exhibited massive inflammatory cell infiltration on the wound surface, indicating that the presence of GA favors the accumulation of early inflammation-related cells at the wound site, promotes their functions and provides certain support for the regulation of early inflammation during wound repair. By day 14, as shown in Figure 6B and F, hematoxylin-eosin staining revealed wide wound gaps in the model and GA groups, whereas CM and ADSCs significantly reduced the gap widths (*P* < 0.01). Notably, GA-CM and GA-ADSCs achieved the narrowest wound gaps and nearly complete closure, with GA-ADSCs exhibiting the highest healing efficacy. Masson’s trichrome staining (Figure 6C and G) showed minimal and disorganized collagen deposition in the model group (31.67 ± 2.26%), while CM (52.52 ± 0.51%) and ADSCs (57.11 ± 2.01%) significantly increased the collagen content (*P* < 0.0001). GA-CM (59.52 ± 0.51%) and GA-ADSCs (69.47 ± 5.59%) exhibited the highest levels, with GA-ADSCs showing densely packed, directionally aligned fibers, indicating collagen regeneration superior to that of GA-CM (*P* < 0.01).

α-SMA immunohistochemistry (Figure 6B and F) was used to assess myofibroblast activation on day 14, a key marker of fibrosis and scar formation. α-SMA staining demonstrated strongest expression in the model group and reduced expression in CM and ADSCs (*P* < 0.05). The GA-CM and GA-ADSC groups further suppressed α-SMA expression, with the effect of GA-ADSC being significantly lower than that of GA-CM (*P* < 0.05). Immunofluorescence staining (Figure 6E, I and J) further assessed angiogenesis (CD31) and fibrosis-related factor expression (TGF-β1). The model and GA groups exhibited weak CD31 signals and high TGF-β1 expression levels, indicating insufficient angiogenesis and fibrosis. CM and ADSCs enhanced CD31 expression and reduced TGF-β1 expression, whereas GA-CM and GA-ADSCs showed the strongest CD31 signals and the lowest TGF-β1 expression. Importantly, TGF-β1 levels were significantly lower in GA-ADSCs than in GA-CM (*P* < 0.05). In summary, our data suggest that both GA-CM and GA-ADSCs effectively promote CDW healing, collagen deposition and neovascularization with the support of GA scaffolds. GA-ADSCs exerted effects on enhancing wound repair and collagen remodeling superior to those of GA-CM, while more effectively suppressing fibrosis- and scar-associated markers (α-SMA and TGF-β1).

### Mechanistic proteomic analysis of GA-ADSCs and GA-CM in chronic diabetic wounds

Proteomic profiling revealed large-scale reprogramming in GA-CM and GA-ADSCs compared with that in GA alone. Compared with GA, both GA-CM and GA-ADSCs triggered extensive proteomic reprogramming with distinct patterns. GA-CM altered the expression of 389 upregulated and 468 downregulated proteins, whereas GA-ADSCs induced broad shifts in the expression of 361 upregulated and 552 downregulated proteins. Despite 23 shared differentially expressed proteins (DEPs) identified under stringent thresholds, heatmap and Venn analyses revealed clear divergence: a set of commonly regulated proteins forming a ‘repair core’, complemented by treatment-specific regulatory modules. Uniquely enriched GA-CM proteins were linked to gene expression and biosynthesis, whereas GA-ADSCs exhibited broad regulation, engaging in immune responses, metabolic remodeling and extracellular matrix (ECM) dynamics. These findings suggest that GA-CM and GA-ADSCs share a common ‘repair core’ while engaging in treatment-specific regulatory modules.

**Figure 7:**
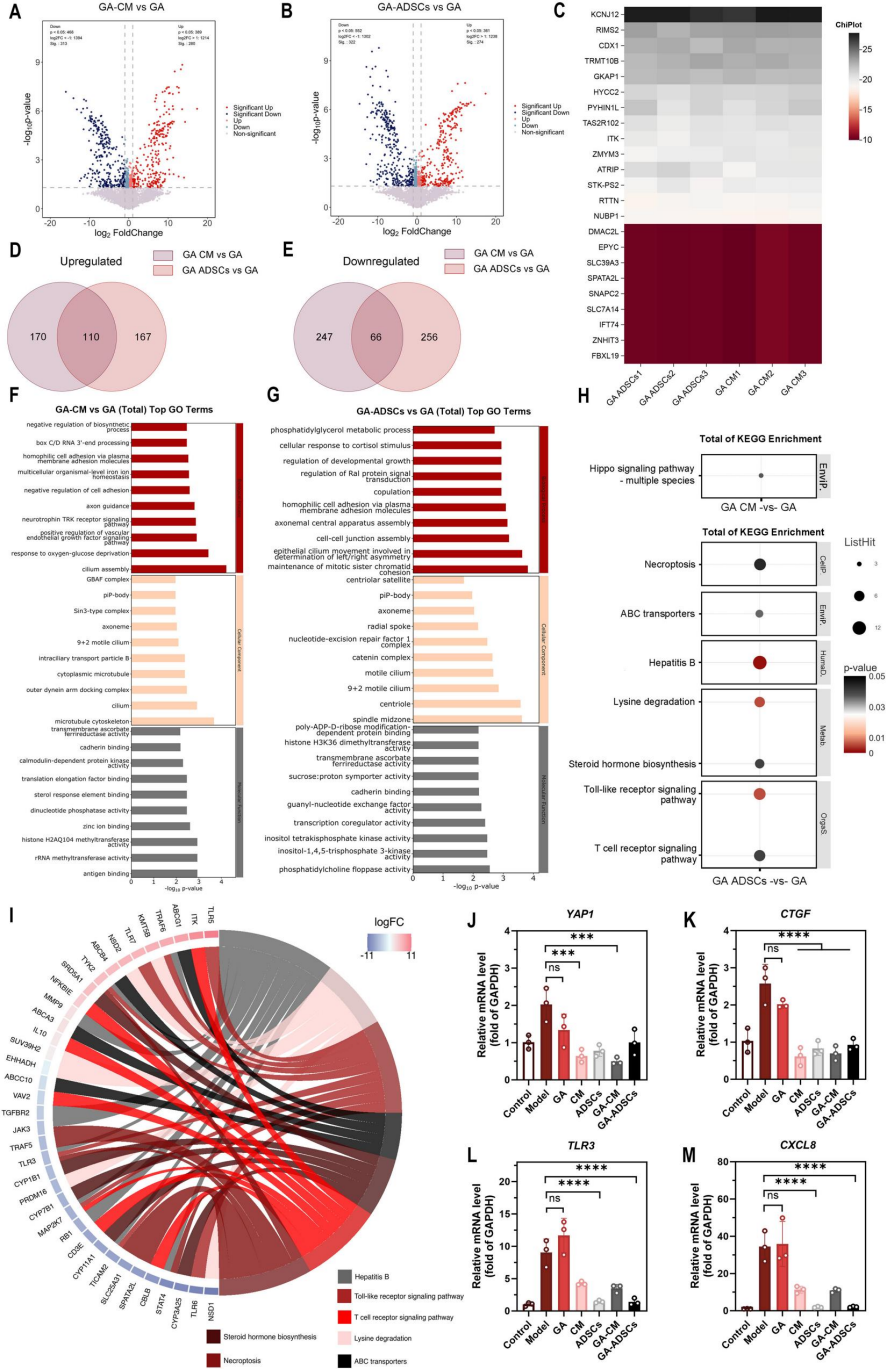
Volcano plots show the upregulated and downregulated expression of proteins in (**A**) GA-CM vs. GA and (**B**) GA-ADSCs vs. GA. (**C**) Heatmap of representative shared DEPs between groups. Venn diagrams of (**D**) upregulated and (**E**) downregulated protein expression in GA-CM vs. GA and GA-ADSCs vs. GA. GO enrichment analysis of DEPs in (**F**) GA-CM vs. GA and (**G**) GA-ADSCs vs. GA. (**H**) KEGG pathway enrichment analysis. (**I**) Chord plot showing the functional associations between DEPs and enriched KEGG pathways. The relative mRNA quantification results of (**J**) YAP, (**K**) CTGF, (**L**) TLR4 and (**M**) CXCL8 genes by qRT-PCR.

Gene ontology enrichment analysis revealed that GA-CM DEPs were primarily enriched in rRNA/histone methyltransferase activity (fold enrichment = 29.29), VEGF- and neurotrophin-related signaling, protein biosynthesis and VEGF-driven angiogenesis and cell migration (Figure 7F). In contrast, GA-ADSCs were significantly enriched in immune stress responses (cortisol, Ral and TLR related), lipid metabolism, signal remodeling (phosphatidylcholine floppase and inositol phosphate kinase activity), chromatin modification and DNA repair (Figure 7G). The Kyoto Encyclopedia of Genes and Genomes pathway analysis confirmed these distinct patterns (Figure 7H). GA-CM was predominantly associated with the Hippo signaling pathway, which is a critical regulator of cell proliferation, migration and angiogenesis, consistent with the growth factor-rich nature of CM. Conversely, GA-ADSCs were prominently involved in multiple pathways related to inflammation and immune regulation (TLR signaling, necroptosis, T-cell receptor signaling and Hepatitis B) as well as cellular metabolism and ECM remodeling (ABC transporters, lysine degradation and steroid biosynthesis). TLR signaling was the most significantly enriched, suggesting a central role in modulating immune responses, inflammation and fibrosis. Furthermore, we summarized the DEPs (*P* < 0.05) involved in the Hippo and TLR signaling pathways for the GA-CM vs GA and GA-ADSCs vs. GA comparisons into Table 2.

**Table 2:** List of representative DEPs enriched in Hippo and Toll-like receptor signaling pathways.

KEGG	Groups	Accession	Protein name	*P*-value
Hippo signaling pathway—multiple species	GA CM-vs-GA	P46938	YAP1	0.00638771
Hippo signaling pathway—multiple species	GA CM-vs-GA	Q8C0V9	FRMD6	0.04621626
Hippo signaling pathway—multiple species	GA CM-vs-GA	E9PVD3	DCHS1	0.00072466
Hippo signaling pathway—multiple species	GA ADSCs-vs-GA	E9PVD3	DCHS1	0.00000057
TLR signaling pathway	GA CM-vs-GA	Q99K90	TAB2	0.00013946
TLR signaling pathway	GA CM-vs-GA	P70434	IRF7	0.00002434
TLR signaling pathway	GA ADSCs-vs-GA	Q9EPW9	TLR6	0.00000322
TLR signaling pathway	GA ADSCs-vs-GA	Q9JLF7	TLR5	0.00000080
TLR signaling pathway	GA ADSCs-vs-GA	Q99MB1	TLR3	0.00013786
TLR signaling pathway	GA ADSCs-vs-GA	Q8BJQ4	TICAM2	0.00000619

To further verify the reliability of the proteomic and pathway results, we performed qRT‑PCR analysis (Figure 7J). Seven experimental groups were established: the control group consisted of untreated HDFs, while the other groups were HDFs induced by LPS and IFN‑α with corresponding interventions. The mRNA expression levels of key genes in the Hippo signaling pathway (YAP, CTGF) and the TLR signaling pathway (TLR3, CXCL8) were detected. The qRT‑PCR results showed that the expression of YAP, CTGF, TLR3 and CXCL8 was significantly increased in the model group compared with the control group. Compared with the model group, the expression of these genes in the GA group was slightly changed without statistical significance (*P* > 0.05). CM and GA-CM significantly reduced the expression of YAP and CTGF (*P* < 0.01), and mildly downregulated the expression of TLR3 and CXCL8. In contrast, GA-ADSCs and ADSCs mildly reduced the expression of YAP and CTGF, and significantly downregulated the expression of TLR3 and CXCL8 (*P* < 0.01).

Collectively, these results demonstrate that GA‑CM and GA‑ADSCs mediate skin wound repair through distinct but complementary mechanisms. GA‑CM mainly acts by regulating the Hippo signaling pathway, thereby suppressing YAP/CTGF-mediated fibroblast activation and excessive ECM deposition, ultimately reducing fibrosis and promoting balanced tissue remodeling. In contrast, GA‑ADSCs primarily exert strong immunomodulatory effects by suppressing the TLR signaling pathway, which helps resolve inflammation, maintain immune homeostasis and support sustained tissue repair. These findings highlight the functional divergence between cell‑free (GA‑CM) and cell‑based (GA‑ADSCs) strategies, and provide a mechanistic rationale for their combined application in the treatment of CDWs.

### Functional implications of DEPs in GA-ADSCs and GA-CM

**Figure 8:**
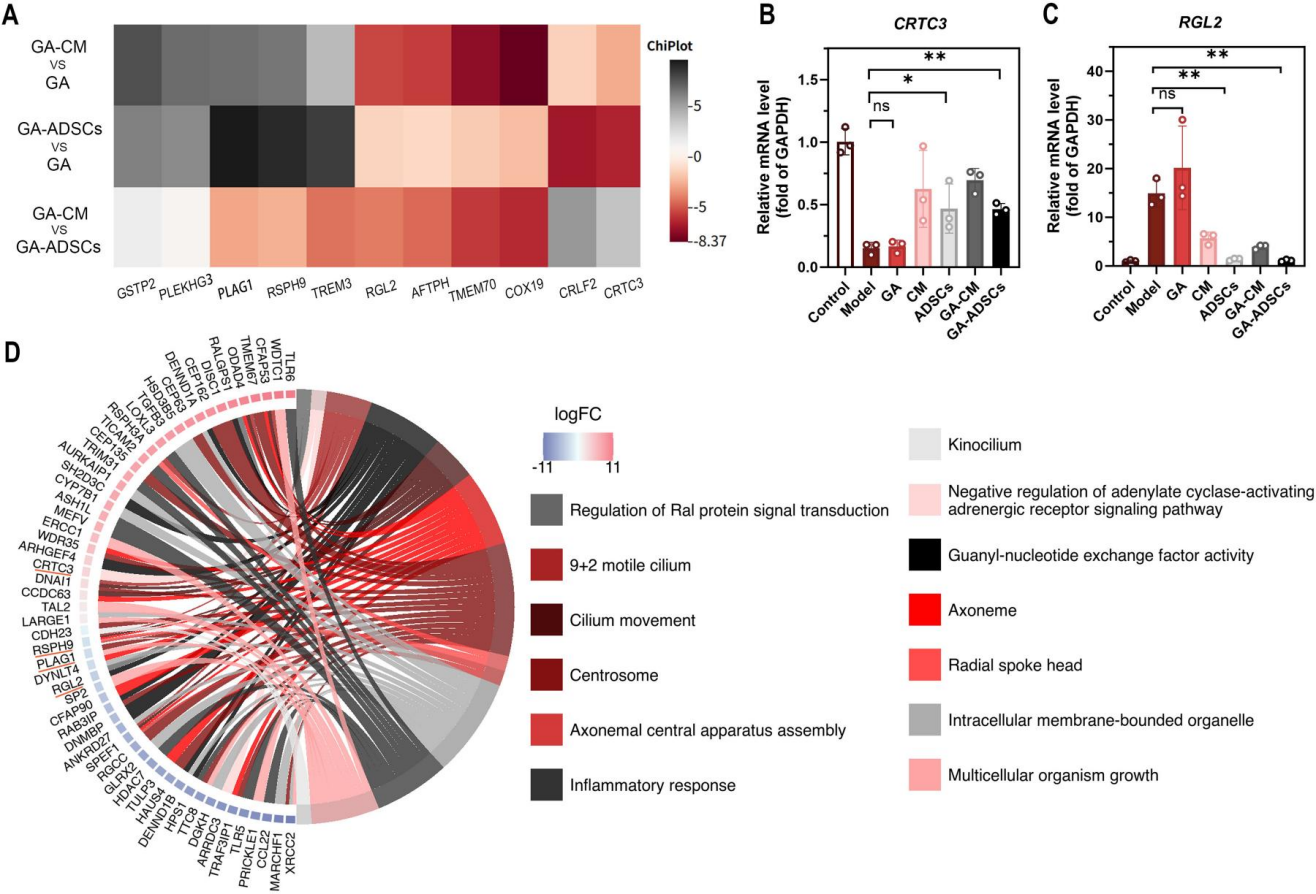
(**A**) Heatmap showing expression patterns of 14 DEPs across the three groups. The relative mRNA quantification results of (**B**) CRTC3 and (**C**) RGL2 genes expression by qRT-PCR. (**D**) Functional enrichment analysis of DEPs highlighting the pathways.

Based on the quantitative proteomics results, we performed functional enrichment analyses of the DEPs in GA-ADSCs vs. GA, GA-CM vs. GA and GA-ADSCs vs. GA-CM to explore their potential roles in wound repair. As shown in Table 3 and Figure 8A, 14 representative DEPs were identified. Among them, the expression of GSTP2, PLEKHG3, RSPH9, TREM3 and PLAG1 was significantly upregulated in GA-ADSCs vs. GA and GA-CM vs. GA, whereas the expression of RGL2, AFTPH, COX19, TMEM70, CRLF2 and CRTC3 was consistently downregulated. Further comparison revealed that GSTP2, PLEKHG3, CRLF2 and CRTC3 were expressed at higher levels in GA-CM than in GA-ADSCs, whereas RSPH9, TREM3, PLAG1, RGL2, AFTPH, COX19 and TMEM70 were more highly expressed in GA-ADSCs than in GA-CM.

**Table 3:** List of representative DEPs in GA-ADSCs vs. GA, GA-CM vs. GA and GA-ADSCs vs. GA-CM.

Protein name	GA CM-vs-GA	GA ADSCs-vs-GA	GA CM-vs-GA ADSCs
GSTP2	Up	Up	Up
PLEKHG3	Up	Up	Up
CRTC3	Up	Up	Up
RSPH9	Up	Up	Down
TREM3	Up	Up	Down
PLAG1	Up	Up	Down
RGL2	Down	Down	Up
CRLF2	Down	Down	Up
AFTPH	Down	Down	Down
COX19	Down	Down	Down
TMEM70	Down	Down	Down

To further verify the reliability of the proteomic results, we performed a qRT-PCR assay to investigate the expression levels of two key DEPs (CRTC3 and RGL2) under different treatment conditions (Figure 8B and C). Specifically, the qRT-PCR results showed that compared with the control group, the expression level of CRTC3 in the model group was significantly decreased (*P* < 0.001). In contrast to the model group, there was no significant change in CRTC3 expression in the GA group (*P* > 0.05), while the expression of CRTC3 was significantly increased in both the CM group and GA-CM group (*P* < 0.05). For RGL2, its expression level in the model group was significantly higher than that in the control group, and there was no significant change in the GA group compared with the model group (*P* > 0.05). However, the expression of RGL2 was significantly decreased in both the ADSCs group and GA-ADSCs group (*P* < 0.01). These qRT-PCR results were highly consistent with the proteomic data, confirming the authenticity of the differential expression of CRTC3 and RGL2, and providing reliable genetic level evidence for subsequent mechanism research.

Functional enrichment analysis revealed distinct mechanistic roles of the representative DEPs (Figure 8D). CRTC3, which is involved in the negative regulation of β-adrenergic receptor signaling, modulates the β-AR–cAMP/PKA axis to enhance angiogenesis, fibroblast proliferation, collagen deposition and macrophage balance. RSPH9, which is associated with the axoneme pathway, regulates primary cilia function and cellular responsiveness to external cues. PLAG1, which is enriched in GA-ADSCs, contributes to centrosome organization and directed cell migration, whereas RGL2 downregulation is associated with reduced inflammatory signaling, influenced keratinocyte/fibroblast migration and improved immune homeostasis during wound healing. Overall, GA-ADSCs and GA-CM modulate distinct protein networks: GA-CM favors growth factor-driven pathways, whereas GA-ADSCs exert stronger effects on immune regulation and metabolic reprogramming—particularly through cilia-associated sensing (RSPH9) and metabolism-related signaling (CRTC3)—than GA-CM. These differences highlight the unique contribution of ADSCs to long-term skin tissue repair and provide new mechanistic insights into chronic wound therapy.

## Discussion

CDWs remain a major clinical challenge owing to the impaired angiogenesis, dysregulated inflammation and reduced functionality of macrophages and fibroblasts under hyperglycemic conditions [30, 31]. Conventional treatments are insufficient, highlighting the urgent need for novel regenerative strategies that integrate biomaterials with advanced biological therapies.

In recent years, bioactive peptides, ADSCs and their CM have become promising interventions for skin wound repair, with distinct characteristics. Bioactive peptides exert precise regulation by targeting specific signaling pathways and have better stability due to simple structure, but show insufficient synergy and limitations in clinical translation (e.g. short *in vivo* half-life). In contrast, ADSCs and CM achieve multi-pathway regulation with significant synergistic effects on wound repair processes, but have weaker targeting specificity, unstable activity and challenges in transportation, storage and immune compatibility [32–36].

In this study, we used a DLP-based 3D bioprinting approach to construct GA hydrogel scaffolds for the delivery of ADSCs or their cell-free CM and systematically compared the distinct mechanisms underlying GA-ADSC and GA-CM efficacy in diabetic wound healing. Centered on ‘structural bionics-functional adaptation’, the 3D-printed structure mimics natural skin microstructure, providing adhesion sites for repair cells and growth factors [37]. Compared with traditional scaffolds, DLP printing enables precise parameter control for a connected pore network (ensuring material exchange and cell growth) and grid-like pores that guide cell directional growth, reduce scars, and enhance tissue mechanical strength [38].

GA scaffolds possess a double-crosslinked interpenetrating polymer network, which can provide mechanical stability, controlled release properties and a biomimetic ECM-like microenvironment, thereby supporting cell viability and maintaining paracrine function. Clarifying the scientific relationship between 3D-printed GA scaffolds, ADSCs and CM, as well as their functional division in wound therapy, is crucial for understanding their therapeutic mechanisms. Verified by *in vitro* cell experiments and *in vivo* animal studies, we clearly distinguished the roles of each component: ADSCs and CM are the core key factors for wound treatment, directly determining the repair outcome; whereas 3D-printed GA scaffolds mainly act as a delivery vehicle to provide auxiliary support and a functional platform for bioactive components.

Specifically, the core value of DLP 3D bioprinting lies in constructing a biomimetic 3D scaffold structure that mimics the natural skin ECM, providing a stable microenvironment for the adhesion, colonization and proliferation of ADSCs. Confocal imaging results confirmed that ADSCs could stably attach to the surface of GA hydrogels, and a larger number of ADSCs were observed inside the scaffold pores with an interlaced growth pattern, fully demonstrating that the 3D porous structure is favorable for cell retention and growth. Meanwhile, GA hydrogels exhibit excellent drug-loading and sustained-release capabilities. After loading ADSCs or CM, they enable the slow and continuous release of bioactive factors, prolong the local action duration and increase the local effective concentration of active components, effectively solving the bottleneck problems of low colonization rate in direct ADSC transplantation and easy loss of CM.

As the core of wound repair, ADSCs and CM undertake critical bioactive regulatory functions. Among them, CM, as the main component of cellular secretions, is rich in various growth factors and bioactive molecules related to wound repair, which can directly regulate inflammation resolution, angiogenesis and epithelialization in wounds. ADSCs further enhance the repair effect through self-proliferation, differentiation and paracrine signaling, and can form a dynamic crosstalk with the wound microenvironment, exerting long-term immunomodulatory and antifibrotic effects.

Our findings showed that both GA-ADSC and GA-CM scaffolds significantly accelerated wound closure, enhanced fibroblast proliferation and migration, and promoted angiogenesis. *In vivo* imaging confirmed that GA hydrogels significantly prolonged retention of ADSCs cells, overcoming the limitations of rapid apoptosis and poor survival associated with direct cell transplantation. In the diabetic mouse model, both the GA-ADSC and GA-CM scaffolds achieved near-complete wound closure by day 14, accompanied by robust collagen deposition and neovascularization. Notably, GA-ADSCs exhibited stronger immunoregulatory and antifibrotic effects than GA-CM, as evidenced by enhanced M2 macrophage polarization, reduced α-SMA expression and downregulation of TGF-β1 expression, indicating that their therapeutic activity is mediated through immune modulation and cell–matrix interactions [39–41]. Although CM displayed relatively weak antifibrotic effects, it was more effective than GA-ADSC in promoting wound closure and angiogenesis, consistent with its growth factor-rich secretome [42, 43].

The key novelty of this study lies in the comparative proteomic and functional analyses of GA-ADSCs and GA-CM. GA-CM was predominantly enriched in the Hippo signaling pathway and VEGF-driven angiogenic processes, consistent with its growth factor-mediated activity in stimulating endothelial activation, vascular sprouting and fibroblast migration. Recent studies have suggested that Hippo signaling via YAP/TAZ effectors regulates multiple skin cell types involved in wound repair, including fibroblasts, keratinocytes and endothelial cells, and plays a pivotal role in diabetic wound healing and collagen remodeling [44, 45]. In contrast, GA-ADSCs activate a broad set of immune- and metabolism-related pathways, with TLR signaling showing the most significant enrichment. TLR signaling in CDWs has been implicated in the regulation of oxidative phosphorylation and energy metabolism in macrophages, thereby influencing M1/M2 polarization, while also modulating skin fibrosis by controlling Col-1 and α-SMA expression to affect myofibroblast differentiation [46, 47]. Moreover, proteomic analysis revealed GA-ADSC-specific regulation of proteins, such as RSPH9 (involved in ciliary signaling) and CRTC3 (linked to energy metabolism and fibroblast proliferation) [44], further highlighting that ADSCs contribute not only through paracrine secretion but also via dynamic crosstalk with the wound microenvironment to achieve sustained, multifaceted tissue repair and scar suppression.

In summary, we demonstrated that DLP 3D-printed GA hydrogel scaffolds provide a robust and multifunctional platform to enhance the therapeutic efficacy of both ADSCs and CM in diabetic wound healing. In this context, CM accelerates wound closure and angiogenesis through Hippo-mediated growth factor signaling, whereas ADSCs exert long-lasting antifibrotic and regenerative benefits via TLR-driven immune regulation and metabolic reprogramming. To further improve the research system and clarify the application boundaries, the next phase of research needs to focus on advancing multiple aspects of work: first, it is necessary to verify the therapeutic boundaries in higher animal immunodeficiency models, which is of great significance for further clarifying the application scope and mechanism of action of GA-ADSCs and GA-CM. In particular, regarding the application boundaries of CM therapy, a key forward-looking consideration is whether its therapeutic efficacy would be weakened in models with severely compromised immunity. As CM exerts its therapeutic effects mainly by regulating the local immune microenvironment and secreting bioactive factors (such as growth factors and cytokines), severe immune impairment may disrupt the normal response of target cells to these bioactive factors, or reduce the ability of the local microenvironment to retain and utilize these factors, thereby potentially attenuating the therapeutic effect of CM. Therefore, future studies should specifically verify the therapeutic effect of GA-CM in higher animal models with severe immune deficiency (e.g. nude mice with severe T/B cell deficiency or models induced by immunosuppressive drugs), compare its efficacy with that in normal immune models and explore the underlying mechanisms (such as changes in the expression of key cytokines or signal pathway activation), which will help to clearly define the applicable population and scenario boundaries of CM therapy. Second, we should focus on optimizing the hydrogel formula, systematically evaluate its long-term safety and functionality and promote the clinical transformation of these two dual strategies (ADSCs and CM). Inspired by the cutting-edge research concepts of degradable intelligent delivery systems and synergistic immunotherapy, future studies can further construct intelligent responsive GA-based hydrogel systems with on-demand drug release, controlled degradation properties or photothermal antibacterial capabilities, so as to achieve precise spatiotemporal regulation during the process of diabetic wound repair [48–51]. At the clinical application level, future research can utilize existing high-sensitivity, amplification-free *in situ* detection technologies to achieve real-time dynamic monitoring of key wound biomarkers (such as VEGF and TGF-β1) [52, 53], which will further promote the precise application of GA-ADSCs and GA-CM in wound repair.

## Conclusion

In this study, we developed DLP-based 3D-printed GA hydrogel scaffolds as multifunctional platforms for the delivery of ADSCs or CM to treat CDWs. Both GA-ADSCs and GA-CM significantly accelerated wound closure and promoted fibroblast proliferation, migration and angiogenesis, while also reducing fibrosis. Comparative proteomic and functional analyses revealed distinct but complementary mechanisms. GA-CM primarily activated Hippo signaling and growth factor-driven angiogenesis to achieve rapid wound closure, whereas GA-ADSCs engaged in TLR signaling and broad immune–metabolic pathways, conferring sustained immunomodulation, antifibrotic remodeling and long-term tissue regeneration. Moreover, DEPs in GA-ADSCs and GA-CM, such as PLEKHG3 and CRTC3, were identified as play functional roles in CDWs. Collectively, this study provides mechanistic insights into the differences between cell-based and cell-free therapies and establishes a scientific basis for advancing stem cell-based strategies in the treatment of CDWs.

## Supplementary Material

rbag095_Supplementary_Data

## References

[1] Hajhosseini B , Gurtner GC, Sen CK. Abstract 48: and at last, the wound is healed… or, is it?! In search of an objective way to predict the recurrence of diabetic foot ulcers. Plast Reconstr Surg – Glob Open 2019;7:34–5.

[2] Baltzis D , Eleftheriadou I, Veves A. Pathogenesis and treatment of impaired wound healing in diabetes mellitus: new insights. Adv Ther 2014;31:817–36. 25069580 10.1007/s12325-014-0140-x

[3] Singh N , Armstrong DG, Lipsky BA. Preventing foot ulcers in patients with diabetes. JAMA 2005;293:217–28. 15644549 10.1001/jama.293.2.217

[4] Ghobril C , Grinstaff MW. The chemistry and engineering of polymeric hydrogel adhesives for wound closure: a tutorial. Chem Soc Rev 2015;44:1820–35. 25649260 10.1039/c4cs00332b

[5] Caplan AI , Dennis JE. Mesenchymal stem cells as trophic mediators. J Cell Biochem 2006;98:1076–84. 16619257 10.1002/jcb.20886

[6] Gimble JM , Katz AJ, Bunnell BA. Adipose-derived stem cells for regenerative medicine. Circ Res 2007;100:1249–60. 17495232 10.1161/01.RES.0000265074.83288.09 PMC5679280

[7] Al-Nbaheen M , Vishnubalaji R, Ali D, Bouslimi A, Al-Jassir F, Megges M, Prigione A, Adjaye J, Kassem M, Aldahmash A. Human stromal (mesenchymal) stem cells from bone marrow, adipose tissue and skin exhibit differences in molecular phenotype and differentiation potential. Stem Cell Rev Rep 2013;9:32–43. 22529014 10.1007/s12015-012-9365-8 PMC3563956

[8] Salibian AA , Widgerow AD, Abrouk M, Evans GR. Stem cells in plastic surgery: a review of current clinical and translational applications. Arch Plast Surg 2013;40:666–75. 24286038 10.5999/aps.2013.40.6.666 PMC3840172

[9] Gimble JM , Guilak F, Bunnell BA. Clinical and preclinical translation of cell-based therapies using adipose tissue-derived cells. Stem Cell Res Ther 2010;1:19. 20587076 10.1186/scrt19 PMC2905095

[10] Strem BM , Hicok KC, Zhu M, Wulur I, Alfonso Z, Schreiber RE, Fraser JK, Hedrick MH. Multipotential differentiation of adipose tissue-derived stem cells. Keio J Med 2005;54:132–41. 16237275 10.2302/kjm.54.132

[11] Chimenti I , Smith RR, Li TS, Gerstenblith G, Messina E, Giacomello A, Marbán E. Relative roles of direct regeneration versus paracrine effects of human cardiosphere-derived cells transplanted into infarcted mice. Circ Res 2010;106:971–80. 20110532 10.1161/CIRCRESAHA.109.210682 PMC4317351

[12] Toma C , Wagner WR, Bowry S, Schwartz A, Villanueva F. Fate of culture-expanded mesenchymal stem cells in the microvasculature: in vivo observations of cell kinetics. Circ Res 2009;104:398–402. 19096027 10.1161/CIRCRESAHA.108.187724 PMC3700384

[13] Ide C , Nakai Y, Nakano N, Seo TB, Yamada Y, Endo K, Noda T, Saito F, Suzuki Y, Fukushima M, Nakatani T. Bone marrow stromal cell transplantation for treatment of sub-acute spinal cord injury in the rat. Brain Res 2010;1332:32–47. 20307513 10.1016/j.brainres.2010.03.043

[14] Solursh M , Meier S. A conditioned medium (CM) factor produced by chondrocytes that promotes their own differentiation. Dev Biol 1973;30:279–89. 4267377 10.1016/0012-1606(73)90089-4

[15] Chen W , Sun Y, Gu X, Cai J, Liu X, Zhang X, Chen J, Hao Y, Chen S. Conditioned medium of human bone marrow-derived stem cells promotes tendon-bone healing of the rotator cuff in a rat model. Biomaterials 2021;271:120714. 33610048 10.1016/j.biomaterials.2021.120714

[16] Huang S , Wu Y, Gao D, Fu X. Paracrine action of mesenchymal stromal cells delivered by microspheres contributes to cutaneous wound healing and prevents scar formation in mice. Cytotherapy 2015;17:922–31. 25939802 10.1016/j.jcyt.2015.03.690

[17] Jeong JH. Adipose stem cells and skin repair. Curr Stem Cell Res Ther 2010;5:137–40. 19941454 10.2174/157488810791268690

[18] Chen L , Tredget EE, Wu PY, Wu Y. Paracrine factors of mesenchymal stem cells recruit macrophages and endothelial lineage cells and enhance wound healing. PLoS One 2008;3:e1886. 18382669 10.1371/journal.pone.0001886 PMC2270908

[19] Rehman J , Traktuev D, Li J, Merfeld-Clauss S, Temm-Grove CJ, Bovenkerk JE, Pell CL, Johnstone BH, Considine RV, March KL. Secretion of angiogenic and antiapoptotic factors by human adipose stromal cells. Circulation 2004;109:1292–8. 14993122 10.1161/01.CIR.0000121425.42966.F1

[20] Rehman J , Considine RV, Bovenkerk JE, Li J, Slavens CA, Jones RM, March KL. Obesity is associated with increased levels of circulating hepatocyte growth factor. J Am Coll Cardiol 2003;41:1408–13. 12706940 10.1016/s0735-1097(03)00231-6

[21] Ebrahimian TG , Pouzoulet F, Squiban C, Buard V, André M, Cousin B, Gourmelon P, Benderitter M, Casteilla L, Tamarat R. Cell therapy based on adipose tissue-derived stromal cells promotes physiological and pathological wound healing. Arterioscler Thromb Vasc Biol 2009;29:503–10. 19201690 10.1161/ATVBAHA.108.178962

[22] Bakadia BM , Qaed Ahmed AA, Lamboni L, Shi Z, Mutu Mukole B, Zheng R, Pierre Mbang M, Zhang B, Gauthier M, Yang G. Engineering homologous platelet-rich plasma, platelet-rich plasma-derived exosomes, and mesenchymal stem cell-derived exosomes-based dual-crosslinked hydrogels as bioactive diabetic wound dressings. Bioact Mater 2023;28:74–94. 37234363 10.1016/j.bioactmat.2023.05.002 PMC10206161

[23] Dong Y , Rodrigues M, Kwon SH, Li X, A S, Brett EA, Elvassore N, Wang W, Gurtner GC. Acceleration of diabetic wound regeneration using an in situ-formed stem-cell-based skin substitute. Adv Healthc Mater 2018;7:e1800432. 30004192 10.1002/adhm.201800432

[24] Cao B , Wang C, Guo P, Zhang Q, Wang C, Sun H, Wen H, Chen X, Wang Y, Wang Y, Huang S, Xue W. Photo-crosslinked enhanced double-network hydrogels based on modified gelatin and oxidized sodium alginate for diabetic wound healing. Int J Biol Macromol 2023;245:125528. 37385313 10.1016/j.ijbiomac.2023.125528

[25] Kurian AG , Singh RK, Patel KD, Lee JH, Kim HW. Multifunctional GelMA platforms with nanomaterials for advanced tissue therapeutics. Bioact Mater 2022;8:267–95. 34541401 10.1016/j.bioactmat.2021.06.027 PMC8424393

[26] Yue K , Trujillo-de Santiago G, Alvarez MM, Tamayol A, Annabi N, Khademhosseini A. Synthesis, properties, and biomedical applications of gelatin methacryloyl (GelMA) hydrogels. Biomaterials 2015;73:254–71. 26414409 10.1016/j.biomaterials.2015.08.045 PMC4610009

[27] Chen Q , Tian X, Fan J, Tong H, Ao Q, Wang X. An interpenetrating alginate/gelatin network for three-dimensional (3D) cell cultures and organ bioprinting. Molecules 2020;25:756. 32050529 10.3390/molecules25030756 PMC7036974

[28] Jorgensen AM , Gorkun A, Mahajan N, Willson K, Clouse C, Jeong CG, Varkey M, Wu M, Walker SJ, Molnar JA, Murphy SV, Lee SJ, Yoo JJ, Soker S, Atala A. Multicellular bioprinted skin facilitates human-like skin architecture in vivo. Sci Transl Med 2023;15:eadf7547. 37792956 10.1126/scitranslmed.adf7547

[29] Liang Y , He J, Guo B. Functional hydrogels as wound dressing to enhance wound healing. ACS Nano 2021;15:12687–722. 34374515 10.1021/acsnano.1c04206

[30] Falanga V. Wound healing and its impairment in the diabetic foot. Lancet 2005;366:1736–43. 16291068 10.1016/S0140-6736(05)67700-8

[31] Brem H , Tomic-Canic M. Cellular and molecular basis of wound healing in diabetes. J Clin Invest 2007;117:1219–22. 17476353 10.1172/JCI32169 PMC1857239

[32] Wang L , Fu Z, Su Y, Yin W, Wang X, Zhao W, Wang J, Li Y, Liu N, Su W, He L, Yin S, Wang Y, Yang X. Cyclic heptapeptide FZ1 acts as an integrin αvβ3 agonist to facilitate diabetic skin wound healing by enhancing angiogenesis. J Med Chem 2025;68:19503–20. 40910702 10.1021/acs.jmedchem.5c01734

[33] Li C , Xiong Y, Fu Z, Ji Y, Yan J, Kong Y, Peng Y, Ru Z, Huang Y, Li Y, Yang Y, He L, Tang J, Wang Y, Yang X. The direct binding of bioactive peptide Andersonin-W1 to TLR4 expedites the healing of diabetic skin wounds. Cell Mol Biol Lett 2024;29:24. 38317065 10.1186/s11658-024-00542-4 PMC10845795

[34] Ru ZQ , Wu YT, Yang CY, Yang YT, Li YJ, Liu M, Peng Y, Yang YL, Wang JY, Jia QY, Li YS, Fu Z, Yang MF, Tang J, Fan Y, Liu CX, Su WR, Liu NX, He L, Wang Y, Yang XW. Ultra-short cyclic peptide Cy (RL-QN15) acts as a TLR4 antagonist to expedite oral ulcer healing. Zool Res 2025;46:1187–202. 41017403 10.24272/j.issn.2095-8137.2025.211 PMC12780494

[35] Wu YT , Ru ZQ, Peng Y, Fu Z, Jia QY, Kang ZJ, Li YS, Huang YB, Yin SG, Guo K, Liu NX, Feng CA, Tang J, Zhang BY, Yang Z, Wang Y, Yang XW. Peptide Cy (RL-QN15) accelerates hair regeneration in diabetic mice by binding to the Frizzled-7 receptor. Zool Res 2024;45:1287–99. 39479995 10.24272/j.issn.2095-8137.2024.134 PMC11668943

[36] Li Y , Jia Q, Liu N, Yin S, Wang J, Ding Y, Yang Y, Peng Y, Ru Z, Zhang S, Be Qi, Sun J, He L, Wang Y, Guo K, Yang X. Peptide RL-QN15 regulates functions of epidermal stem cells to accelerate skin wound regeneration via the FZD8/β-catenin axis. Exploration 2025;20240090. 10.1002/EXP.20240090 PMC13317571 42382691

[37] Hofmann E , Schwarz A, Fink J, Kamolz LP, Kotzbeck P. Modelling the complexity of human skin in vitro. Biomedicines 2023;11:794. 36979772 10.3390/biomedicines11030794 PMC10045055

[38] Li J , Liu X, Tao W, Li Y, Du Y, Zhang S. Micropatterned composite membrane guides oriented cell growth and vascularization for accelerating wound healing. Regen Biomater 2023;10:rbac108. 36683746 10.1093/rb/rbac108 PMC9847515

[39] Louiselle AE , Niemiec SM, Zgheib C, Liechty KW. Macrophage polarization and diabetic wound healing. Transl Res 2021;236:109–16. 34089902 10.1016/j.trsl.2021.05.006

[40] Mantovani A , Biswas SK, Galdiero MR, Sica A, Locati M. Macrophage plasticity and polarization in tissue repair and remodelling. J Pathol 2013;229:176–85. 23096265 10.1002/path.4133

[41] Krzyszczyk P , Schloss R, Palmer A, Berthiaume F. The role of macrophages in acute and chronic wound healing and interventions to promote pro-wound healing phenotypes. Front Physiol 2018;9:419. 29765329 10.3389/fphys.2018.00419 PMC5938667

[42] Rong X , Chu W, Zhang H, Wang Y, Qi X, Zhang G, Wang Y, Li C. Antler stem cell-conditioned medium stimulates regenerative wound healing in rats. Stem Cell Res Ther 2019;10:326. 31744537 10.1186/s13287-019-1457-9 PMC6862758

[43] Zhang C , Wang T, Zhang L, Chen P, Tang S, Chen A, Li M, Peng G, Gao H, Weng H, Zhang H, Li S, Chen J, Chen L, Chen X. Combination of lyophilized adipose-derived stem cell concentrated conditioned medium and polysaccharide hydrogel in the inhibition of hypertrophic scarring. Stem Cell Res Ther 2021;12:23. 33413617 10.1186/s13287-020-02061-3 PMC7792059

[44] Schmidt A , von Woedtke T, Weltmann KD, Bekeschus S. Yap/TAZ, beta-catenin, and TGFb pathway activation in medical plasma-induced wound healing in diabetic mice. J Adv Res 2025;72:387–400. 38986808 10.1016/j.jare.2024.07.004 PMC12147638

[45] Schlegelmilch K , Mohseni M, Kirak O, Pruszak J, Rodriguez JR, Zhou D, Kreger BT, Vasioukhin V, Avruch J, Brummelkamp TR, Camargo FD. Yap1 acts downstream of α-catenin to control epidermal proliferation. Cell 2011;144:782–95. 21376238 10.1016/j.cell.2011.02.031 PMC3237196

[46] Bhattacharyya S , Tamaki Z, Wang W, Hinchcliff M, Hoover P, Getsios S, White ES, Varga J. FibronectinEDA promotes chronic cutaneous fibrosis through Toll-like receptor signaling. Sci Transl Med 2014;6:232ra50. 10.1126/scitranslmed.3008264 PMC4414050 24739758

[47] Lu Z , Tan K, Xiang S, Zhang Y, Luo F, Liu X, Zhao X, Ouyang L. Peptide loaded self-healing hydrogel promotes diabetic skin wound healing through macrophage orchestration and inflammation inhibition. Mater Today Bio 2025;32:101690. 10.1016/j.mtbio.2025.101690 PMC11986612 40225136

[48] Xie Z , Duo Y, Fan T, Zhu Y, Feng S, Li C, Guo H, Ge Y, Ahmed S, Huang W, Liu H, Qi L, Guo R, Li D, Prasad PN, Zhang H. Light-induced tumor theranostics based on chemical-exfoliated borophene. Light Sci Appl 2022;11:324. 36369148 10.1038/s41377-022-00980-9 PMC9652458

[49] Xie Z , Peng M, Lu R, Meng X, Liang W, Li Z, Qiu M, Zhang B, Nie G, Xie N, Zhang H, Prasad PN. Black phosphorus-based photothermal therapy with aCD47-mediated immune checkpoint blockade for enhanced cancer immunotherapy. Light Sci Appl 2020;9:161. 33014356 10.1038/s41377-020-00388-3 PMC7492464

[50] Xie Z , Xing C, Huang W, Fan T, Li Z, Zhao J, Xiang Y, Guo Z, Li J, Yang Z, Dong B, Qu J, Fan D, Zhang H. Ultrathin 2D nonlayered tellurium nanosheets: facile liquid-phase exfoliation, characterization, and photoresponse with high performance and enhanced stability. Adv Funct Mater 2018;28:1705833.

[51] Liu Y , Bhattarai P, Dai Z, Chen X. Photothermal therapy and photoacoustic imaging via nanotheranostics in fighting cancer. Chem Soc Rev 2019;48:2053–108. 30259015 10.1039/c8cs00618k PMC6437026

[52] Chen Z , Li J, Li T, Fan T, Meng C, Li C, Kang J, Chai L, Hao Y, Tang Y, Al-Hartomy OA, Wageh S, Al-Sehemi AG, Luo Z, Yu J, Shao Y, Li D, Feng S, Liu WJ, He Y, Ma X, Xie Z, Zhang H. A CRISPR/Cas12a-empowered surface plasmon resonance platform for rapid and specific diagnosis of the Omicron variant of SARS-CoV-2. Natl Sci Rev 2022;9:nwac104. 35992231 10.1093/nsr/nwac104 PMC9385456

[53] Kang D , Liu Z, Qian C, Huang J, Zhou Y, Mao X, Qu Q, Liu B, Wang J, Hu Z, Miao Y. 3D bioprinting of a gelatin-alginate hydrogel for tissue-engineered hair follicle regeneration. Acta Biomater 2023;165:19–30. 35288311 10.1016/j.actbio.2022.03.011

