# Supplementary material for: Comparative regenerative mechanisms of adipose-derived mesenchymal stem cell- and conditioned medium-loaded three-dimensional bioprinted hydrogels in chronic diabetic wounds
Source: Regen Biomater. 2026 May 13;13:rbag095. doi: 10.1093/rb/rbag095 (PMC13283645; doi:10.1093/rb/rbag095)
Supplement: rbag095_Supplementary_Data [file rbag095_supplementary_data.docx]

Supplementary Information

Comparative Regenerative Mechanisms of Adipose-Derived Mesenchymal Stem Cell- and Conditioned Medium-Loaded Three-dimensional Bioprinted Hydrogels in Chronic Diabetic Wounds

This file includes:

Supplenmtary figures

Figures S1 to S10

Supplenmtary figures


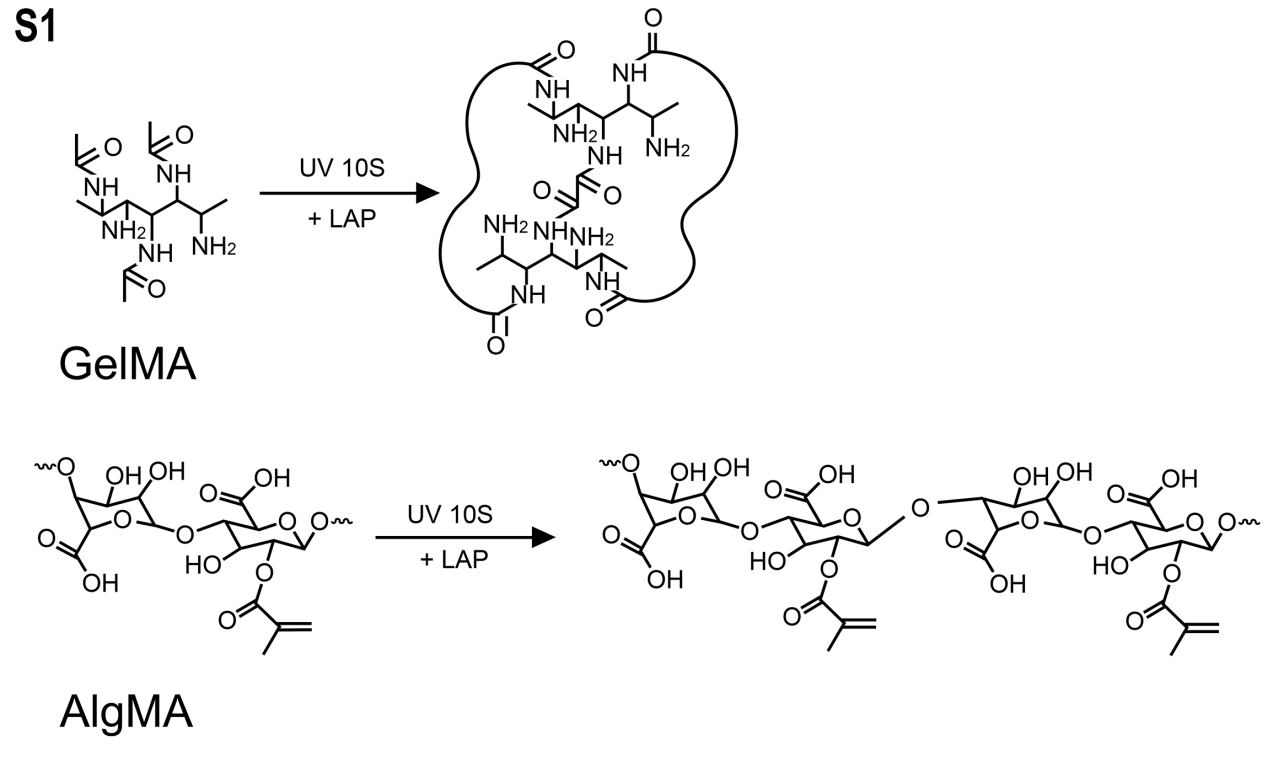


**Figure S1.** Schematic illustration of the photocrosslinking mechanism of GelMA and AlgMA.

**
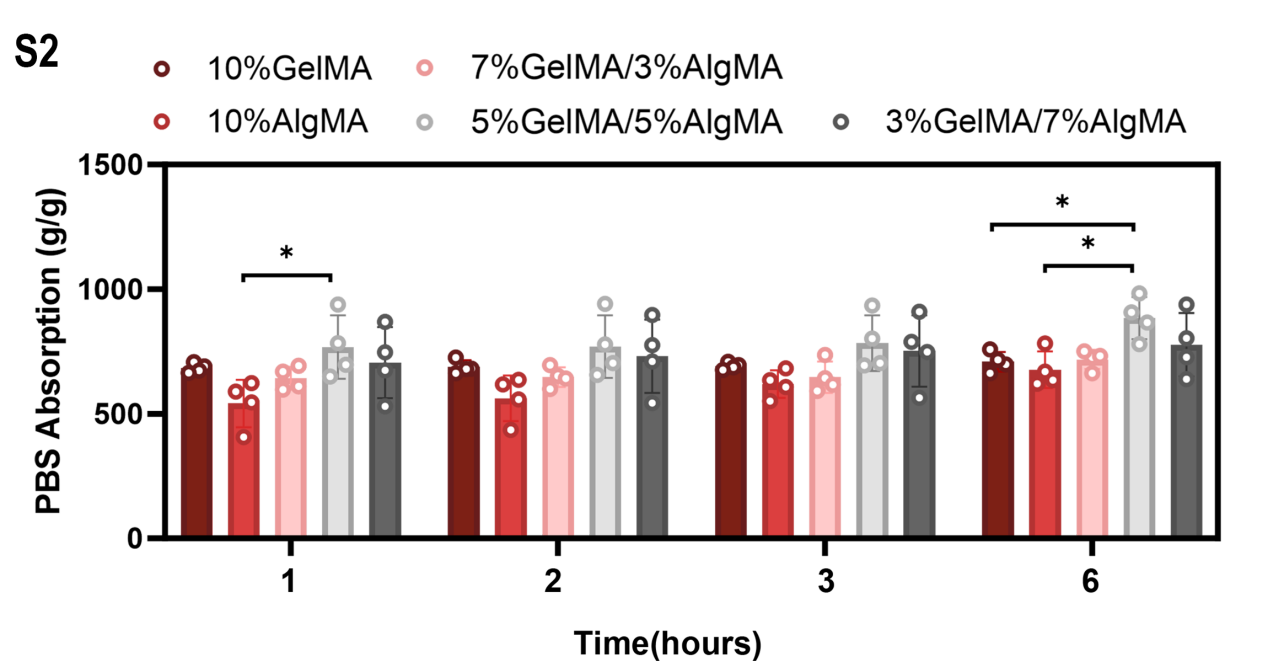
**

**Figure S2.** Swelling ratio analysis of hydrogels with different compositions.

Swelling properties of 10% GelMA group, 10% AlgMA group, 7% GelMA/3% AlgMA group, 5% GelMA/5% AlgMA group, and 3% GelMA/7% AlgMA group were quantitatively analyzed and statistically compared.


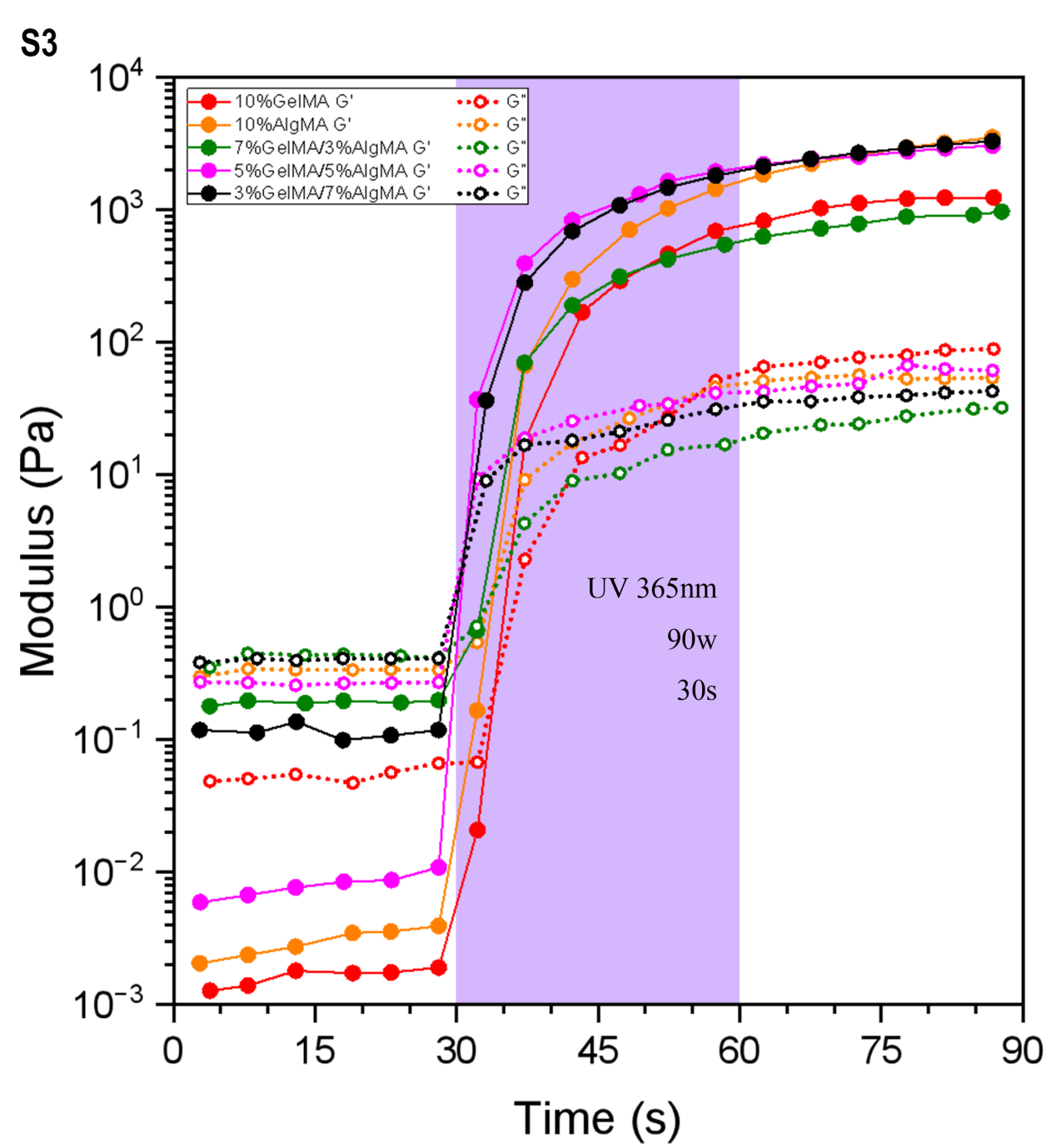


**Figure S3.** Rheological analysis of different hydrogel groups under UV irradiation.

Rheological properties of 10% GelMA group, 10% AlgMA group, 7% GelMA/3% AlgMA group, 5% GelMA/5% AlgMA group, and 3% GelMA/7% AlgMA group were characterized and compared.


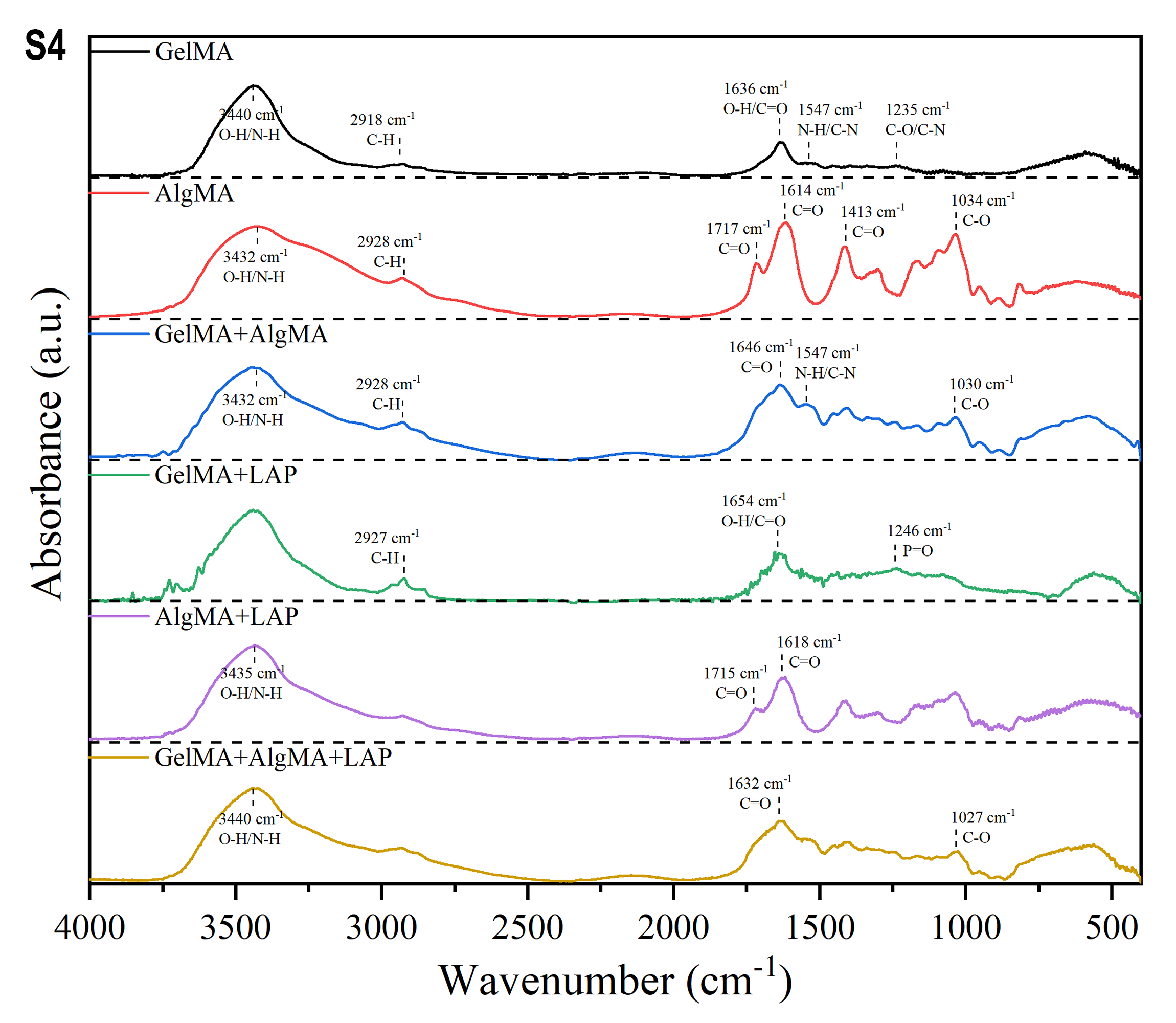


**Figure S4.** FTIR spectra of GelMA, AlgMA, and their composite hydrogels with or without LAP.

Fourier transform infrared (FTIR) spectra were recorded to characterize the chemical structures of GelMA, AlgMA, and their composite hydrogels in the presence or absence of LAP.


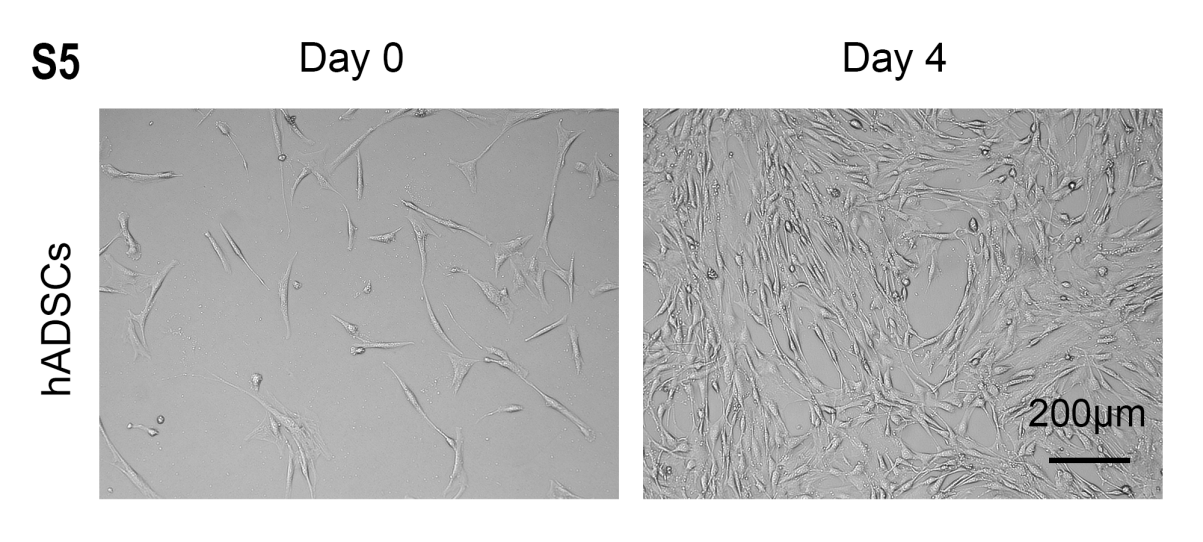


Figure S5. Optical microscope images of ADSCs at day 0 and day 4.

Adipose-derived stem cells (ADSCs) were observed and photographed under an optical microscope on day 0 and day 4 of culture.


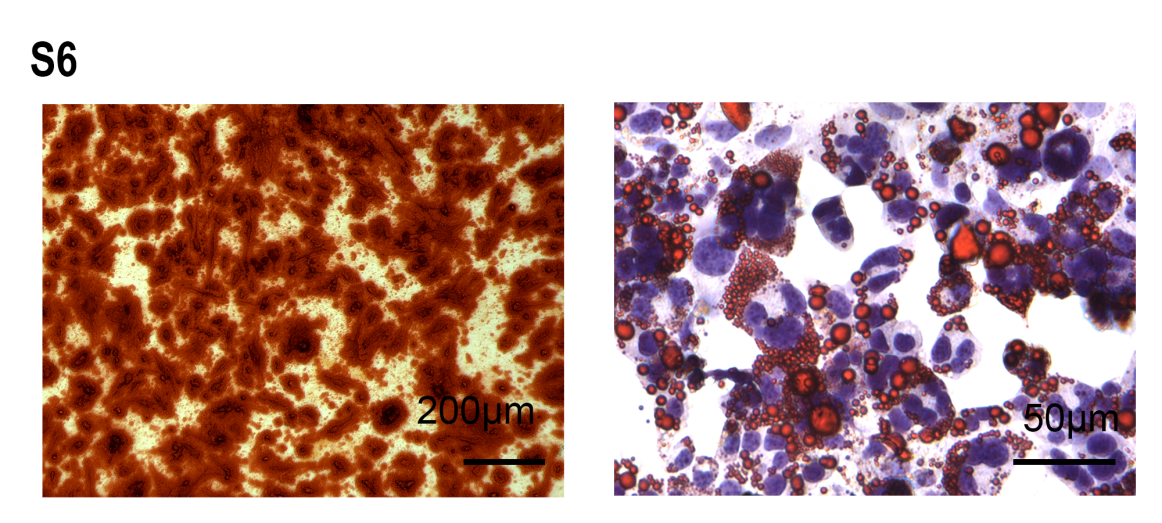


**Figure S6.** Multilineage differentiation of ADSCs confirmed by Oil Red O and Alizarin Red S staining.


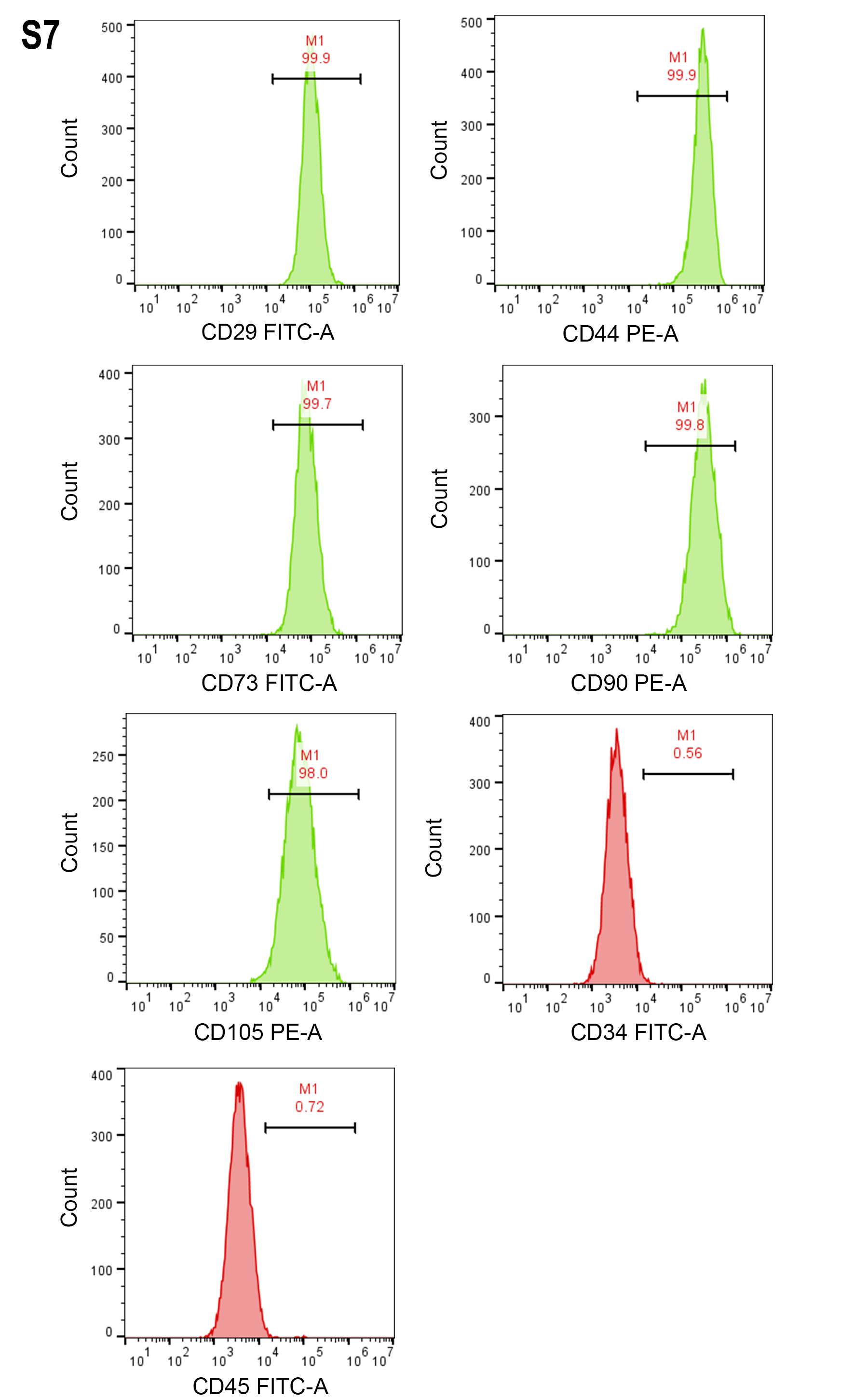


**Figure S7.** Flow cytometry identification of markers CD29, CD44, CD73, CD90, CD105, CD34 and CD45.


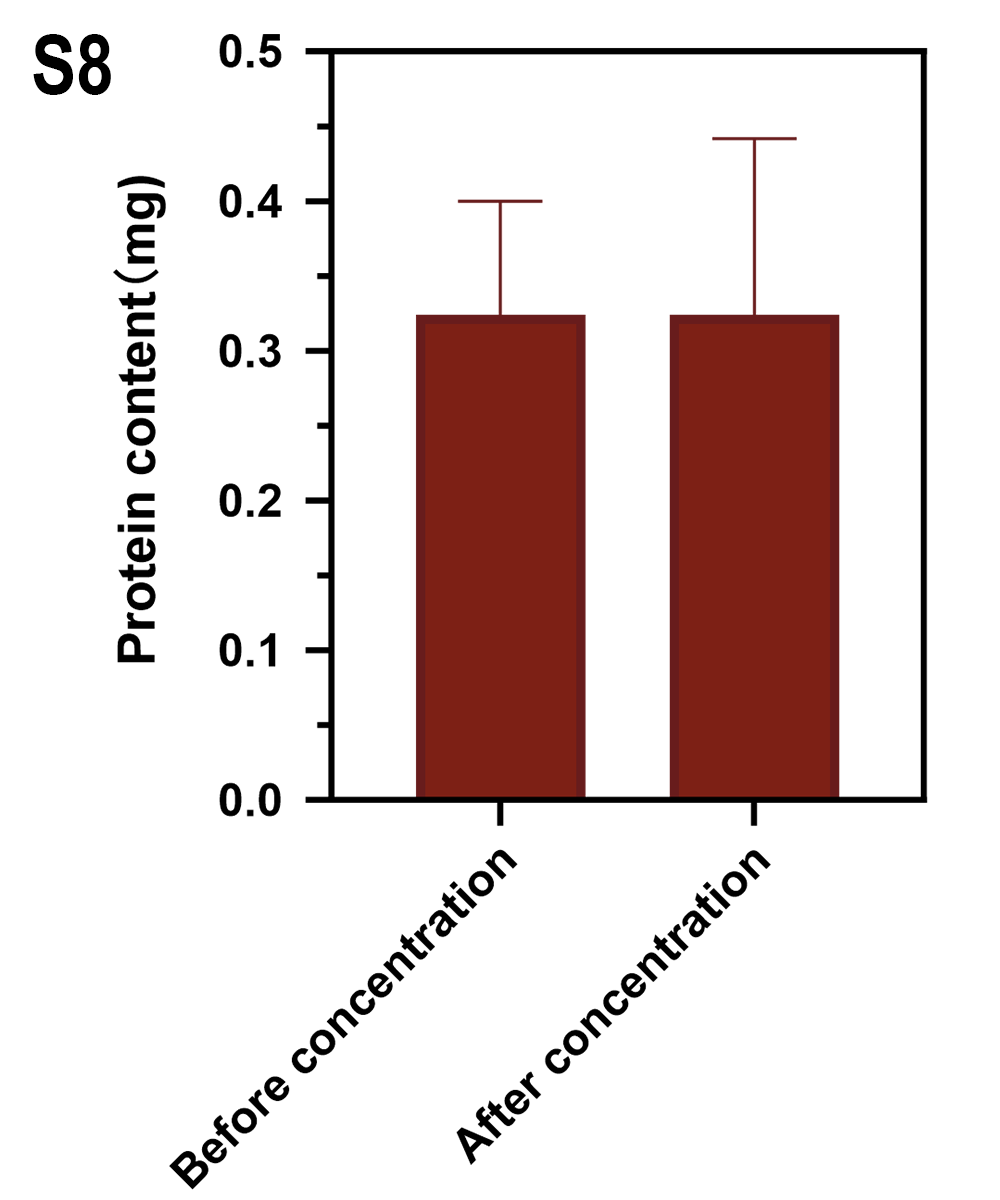


**Figure S8.** Protein content of Conditioned medium (CM) before and after concentration.

CM was obtained by culturing 15×10^5^ ADSCs for 48 hours, and the protein content was measured before and after concentration.


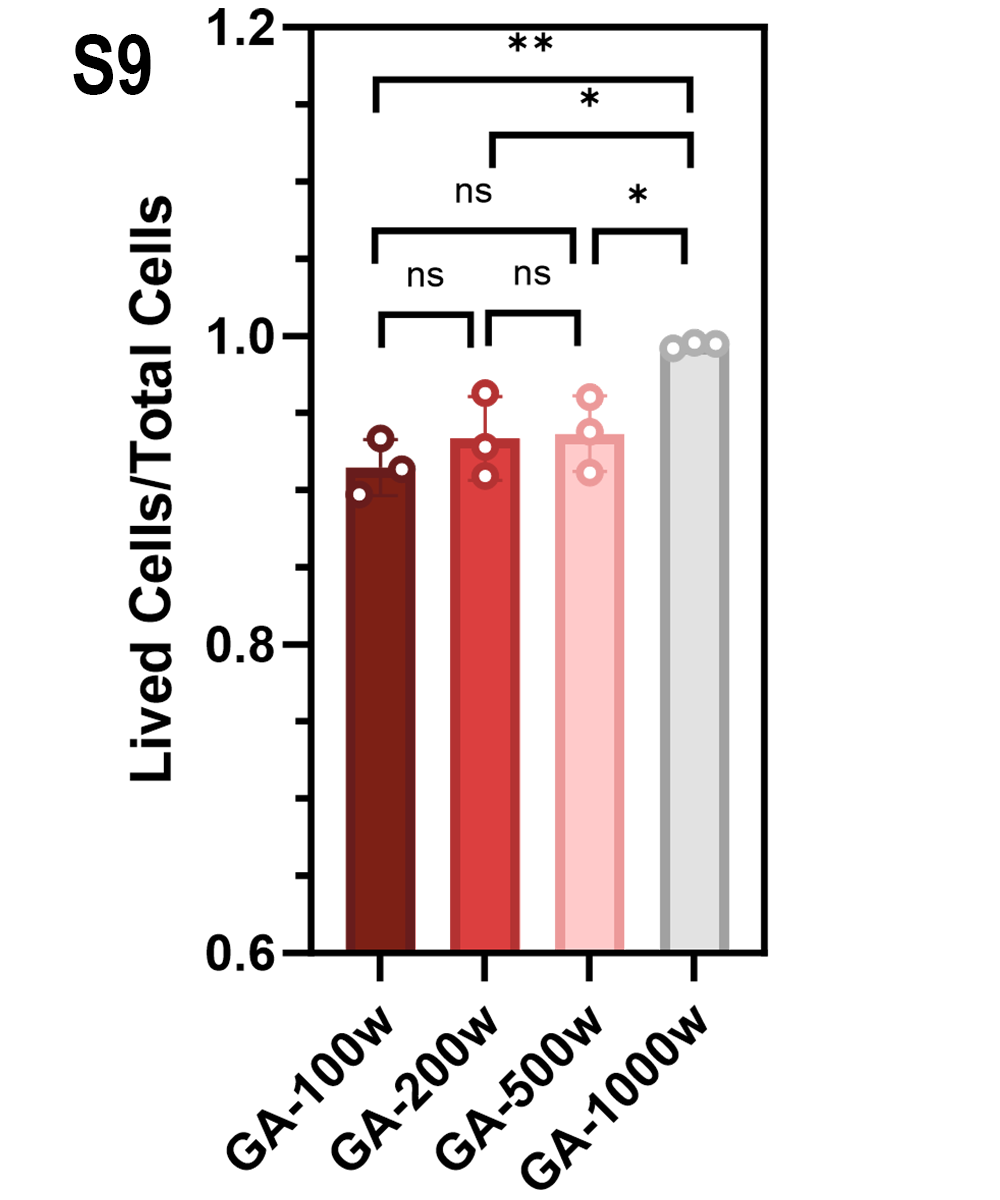


**Figure S9.** Live/dead staining of ADSCs encapsulated in GA scaffolds at different seeding densities.


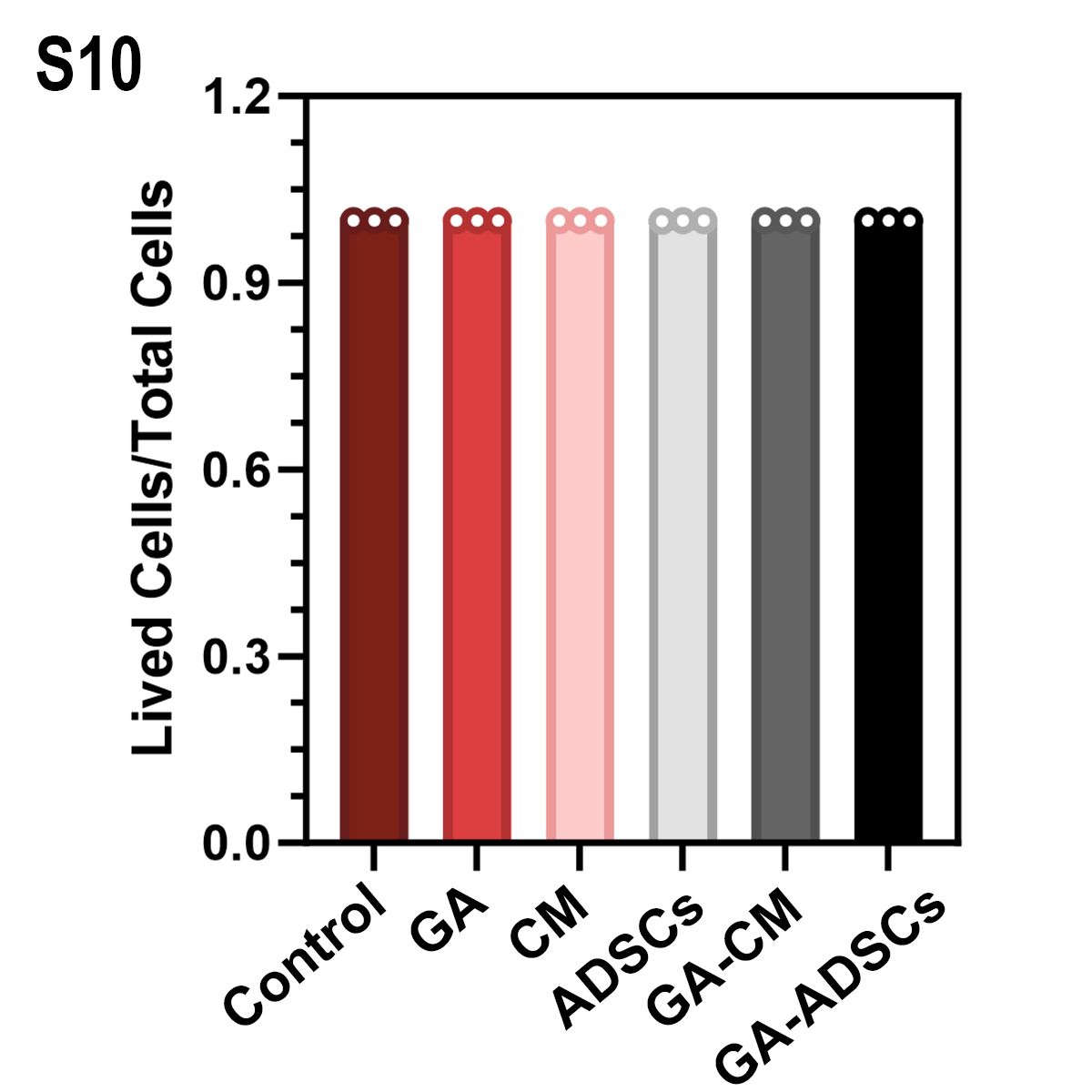


**Figure S10.** Live/dead staining of HDFs co-cultured with different hydrogel groups ( Control, GA, CM, ADSCs, GA-CM, and GA-ADSCs).
